# Mesodermal ALK5 controls lung myofibroblast versus lipofibroblast cell fate

**DOI:** 10.1186/s12915-016-0242-9

**Published:** 2016-03-16

**Authors:** Aimin Li, Shudong Ma, Susan M. Smith, Matt K. Lee, Ashley Fischer, Zea Borok, Saverio Bellusci, Changgong Li, Parviz Minoo

**Affiliations:** Division of Newborn Medicine, Department of Pediatrics, LAC+USC Medical Center and Childrens Hospital Los Angeles, Keck School of Medicine of USC, Los Angeles, CA 90033 USA; Will Rogers Institute Pulmonary Research Center, Division of Pulmonary, Critical Care and Sleep Medicine, Department of Medicine, Keck School of Medicine of USC, Los Angeles, CA 90033 USA; Hastings Center for Pulmonary Research, Keck School of Medicine of USC, Los Angeles, CA 90033 USA; Excellence Cluster Cardio Pulmonary System, University Justus Liebig Giessen, Giessen, 39352 Germany; Institute of Fundamental Medicine and Biology, Kazan Federal University, Kremlevskaya St 18, Kazan, 420008 Russia

**Keywords:** Lipofibroblast, Lung development, Mesoderm, Myofibroblast, *Pdgfrα*, *Pparβ*, *Zfp423*

## Abstract

**Background:**

Epithelial-mesenchymal cross talk is centerpiece in the development of many branched organs, including the lungs. The embryonic lung mesoderm provides instructional information not only for lung architectural development, but also for patterning, commitment and differentiation of its many highly specialized cell types. The mesoderm also serves as a reservoir of progenitors for generation of differentiated mesenchymal cell types that include *αSMA-*expressing fibroblasts, lipofibroblasts, endothelial cells and others. Transforming Growth Factor β (TGFβ) is a key signaling pathway in epithelial-mesenchymal cross talk. Using a cre-loxP approach we have elucidated the role of the TGFβ type I receptor tyrosine kinase, ALK5, in epithelial-mesenchymal cross talk during lung morphogenesis.

**Results:**

Targeted early inactivation of *Alk5* in mesodermal progenitors caused abnormal development and maturation of the lung that included reduced physical size of the sub-mesothelial mesoderm, an established source of specific mesodermal progenitors. Abrogation of mesodermal ALK5-mediated signaling also inhibited differentiation of cell populations in the epithelial and endothelial lineages. Importantly, *Alk5* mutant lungs contained a reduced number of αSMA^pos^ cells and correspondingly increased lipofibroblasts. Elucidation of the underlying mechanisms revealed that through direct and indirect modulation of target signaling pathways and transcription factors, including PDGFRα, PPARγ, PRRX1, and ZFP423, ALK5-mediated TGFβ controls a process that regulates the commitment and differentiation of αSMA^pos^ versus lipofibroblast cell populations during lung development.

**Conclusion:**

ALK5-mediated TGFβ signaling controls an early pathway that regulates the commitment and differentiation of αSMA^pos^ versus LIF cell lineages during lung development.

**Electronic supplementary material:**

The online version of this article (doi:10.1186/s12915-016-0242-9) contains supplementary material, which is available to authorized users.

## Background

In mammals, the anterior foregut invades the surrounding splanchnic mesoderm to form the primordial lung. During embryogenesis, this bilayered structure undergoes a series of highly orchestrated morphogenetic steps to form a complex respiratory organ purported to include over 40 specialized cell varieties. Elucidating the mechanisms that govern commitment and differentiation of these cell types has been a major challenge. The bulk of the cell types studied has been of endodermal origin. Much less is known about the regulatory mechanisms that control the ontogeny and differentiation of the mesodermally-derived cell varieties.

In very broad terms, the lung mesenchyme can be divided into two histologically distinct cell populations that are detectable as early as E13.5 [1]. The ‘sub-epithelial mesenchymal’ cells wrap around the epithelial ducts, while the ‘sub-mesothelial mesenchymal’ (SMM) cells inhabit the area between the mesothelium and the sub-epithelial mesenchymal cells [1, 2]. SMM is a major site of *Fgf10* expression. We and others have shown that *Fgf10*^*pos*^ cells contribute, but are not the sole source of smooth muscle (SM) cells and lipofibroblasts (LIFs) [2, 3]. Viewed from the perspective of gene expression, mesodermal derivatives can be simply grouped into two molecularly defined cell populations; the αSMA^pos^ and αSMA^neg^ groups. The primary αSMA^pos^ group comprises the fibroblasts in the peribronchial (airway) and perivascular SM layers as well as interstitial αSMA^pos^ myofibroblasts. Notably, the latter cells attain αSMA^pos^ status at different times during lung development. While airway and perivascular SM cells are αSMA^pos^ as early as E11.5, interstitial fibroblasts begin to display αSMA only in mid to late gestation. With that caveat in mind, in the present study we have opted to use the term ‘αSMA^pos^ cells’ in a ‘broad stroke’ to conveniently refer collectively to all cells that express this marker and not solely the ‘interstitial myofibroblasts’ noted routinely by other investigators.

Generation of mesodermal cell diversity occurs concurrently with the structural development of the lung. A central player is the reciprocal communication known as epithelial-mesenchymal interactions that occur between the foregut endoderm and the lateral plate mesoderm-derived splanchnic mesenchyme. This process works on a ‘signaling, transcription factors, signaling’ algorithm [4–6]. Additional integral components include the extracellular matrix, structural proteins and differentiation-specific proteins. A major signaling pathway in the lung and other mammalian organs is the transforming growth factor beta (TGFβ) family of secreted polypeptides.

The significance of TGFβ signaling during development and disease can be hardly overstated. TGFβ is the prototype of a family of secreted dimeric peptide growth factors that includes the TGFβs, activins, inhibins, and bone morphogenetic proteins [7]. In vertebrates, TGFβ regulates key processes in stem cell maintenance, organogenesis, wound healing, and homeostasis. Given this broad range of activity, it is not surprising that TGFβ dysregulation results in a spectrum of pathologies ranging from cancer to pulmonary fibrosis.

The TGFβ machinery has many "moving parts. The ligands are produced as ‘latent’ peptides. Upon secretion and activation, all three TGFβs signal by engaging a specific receptor, composed of two related transmembrane serine/threonine kinases, called the type I and type II TGFβ receptors (TβR1, or ALK5 and TβR2) [8]. The basic mechanism of receptor activation involves binding of the ligand to TβR2, followed by recruitment of ALK5. Recruitment triggers ALK5 kinase activity, transducing the signal by phosphorylating and activating members of the SMAD family of transcription factors [8].

In various tissues, TGFβ response is remarkably cell type and context dependent. It is equally true that the downstream effects of TGFβ are transduced not simply via a single target, but may involve multiple nodes. Given the multicomponent nature of the pathway, there is a wide spectrum of versatility and selectivity in TGFβ biologic functions. Some are ostensibly paradoxical. For example, TGFβ acts as both a cancer promoter and suppressor [9]. Selective utilization of receptors is a potential mechanism for generating versatility in TGFβ function. TGFβ expression is thought to be ubiquitous throughout the lung with both endodermal and mesodermal cells displaying ALK5 and TβR2. Elucidating the function of each receptor in specific cell types and in particular in endodermal versus mesodermal cells of the lung is a necessary step towards unlocking the precise roles of TGFβ in development and disease. Attempts to define the specific role played by each of the receptors using targeted germline deletions of either *Tβr2* or *Alk5* have not been fully successful due to early embryonic lethality [10, 11]. Using conditional inactivation, we have shown that lack of mesodermal-specific TβR2 causes embryonic lethality, while endodermal *Tβr2* inactivation is not only tolerated and viable, but protects against experimentally induced fibrosis and bronchopulmonary dysplasia [12, 13]. A systematic approach involving abrogation of each receptor on a cell-type specific basis has the promise of revealing the precise roles of each receptor during embryonic development.

In this study, *Alk5* was specifically inactivated in the early embryonic multipotential mesoderm that is the origin of lung mesodermal progenitors. *Alk5*^*Dermo1*^ mice displayed multiple abnormalities and postnatal lethality. Their lungs were structurally immature (pulmonary hypoplasia), characterized by thickened mesenchymal walls around significantly reduced alveolar spaces. Importantly, the studies revealed a novel role for ALK5-mediated TGFβ signaling that regulates the balance between αSMA^pos^ and LIF cell fate commitment and differentiation during lung development. The precise balance between these key cell populations is critical to normal lung development and its disruption underlies the pathobiology of serious pulmonary disorders.

## Results

### Mesodermal-specific *Alk5* inactivation

The localization and efficiency of LoxP-dependent excision in *Dermo1-cre* (aka, *Twist2-cre*) mouse lungs has been previously reported [14, 15]. *Dermo1*-driven, *Cre*-mediated recombination occurs early and exclusively throughout the tracheal and pulmonary mesoderm, but not in the epithelium (Additional file 1A–C). Accordingly, we generated triple transgenic *Dermo1-cre;Alk5*^*flox/flox*^*;mTmG* mice (Methods) in which exon 3 of the *Alk5* gene is excised in early pulmonary mesodermal progenitors (Additional file 1D). Over 800 fetuses at various embryonic stages were genotyped and analyzed (Additional file 1E).

Heterozygous *Alk5*^*flox/wt*^*;Dermo1-Cre;mTmG* mice were born alive and had no discernable abnormalities in development, growth, or reproduction. Homozygous *Alk5*^*flox/flox*^*;Dermo1-Cre;mTmG* (hereafter *Alk5*^*Dermo1*^) embryos examined at E11.5 showed no obvious morphological defects. Defects in body-wall closure in *Alk5*^*Dermo1*^ embryos, as previously reported [16], were evident at E12.5 (data not shown). Embryonic lethality occurred between E14.5 and E18.5. Occasionally, mutant embryos with a less severe phenotype survived to birth but died immediately afterwards.

To confirm deletion of *Alk5* from the pulmonary mesoderm, expression of ALK5, phospho-SMAD2 (p-SMAD2), and PAI-1 were assessed by immunoblotting. The latter is expressed by mesenchymal cells in response to canonical TGFβ signaling. Compared to controls, ALK5 was decreased by nearly 85 % (0.15 ± 0.013), p-SMAD2 by 73 % (0.23 ± 0.02), and PAI-1 expression reduced by 83 % (0.17 ± 0.05) (Additional file 1F). Immunohistochemistry (IHC) for ALK5 and PAI-1 on E15.5 lung sections confirmed decreased mesenchymal and intact epithelial ALK5 immunoreactivity (Additional file 1G–J). While these results confirm abrogation of canonical TGFβ signaling, the source of the residual activity can be certainly attributed to intact signaling in the lung epithelium.

### Lung immaturity and blocked epithelial and endothelial cell differentiation in *Alk5*^*Dermo1*^ lungs

The lungs of E18.5, *Alk5*^*Dermo1*^ embryos were smaller and poorly expanded, but the number of lobes appeared normal (Additional file 2A–D and data not shown). The alveolar diameters were decreased and septal thicknesses increased relative to control lungs (Additional file 2E–J). Glycogen stores, which are an epithelial marker of immature lungs [17], were also increased in *Alk5*^*Dermo1*^ lungs (Additional file 2G,J). IHC using antibodies against acetylated tubulin, CC10, pro-SPC, and T1a (specific to ciliated cells, Clara cells, alveolar epithelial type II cells, and alveolar epithelial type 1 cells, respectively) showed reduced differentiation of all four major epithelial cell types in E18.5 *Alk5*^*Dermo1*^ lungs (Additional file 3A–H). These findings were also examined by quantitative PCR (Q-PCR) (Additional file 3I). Cumulatively, these results suggest that lung maturation and epithelial cell differentiation are delayed by mesenchymal *Alk5* inactivation.

The lung mesoderm serves as the origin of endothelial cells that form the pulmonary vasculature. *Flk1*, expressed by vascular endothelial progenitors, was reduced in E13.5 *Alk5*^*Dermo1*^ lungs as was the fraction of CD34^pos^ cells (Additional file 3J–M). As lung development progressed, PECAM1, a vascular endothelial differentiation marker, was also reduced. The distal capillary plexus adjacent to the airway epithelium was less dense compared to age-matched controls (Additional file 3N–Q). However, the large blood vessels were intact (Additional file 3P,Q, Arrows). Q-PCR of multiple vascular markers showed that in *Alk5*^*Dermo1*^ lungs, *Pecam-1* mRNA was 0.58 ± 0.19 (*P* <0.05), *Flk1* was 0.63 ± 0.18 (*P* <0.05), and *Flt-1*was 0.67 ± 0.15 (*P* <0.05) of controls (Additional file 3R). However, mutant and control lungs had similar *Flt-4* (0.83 ± 0.23, *P* >0.05) and *Vegfa* (0.78 ± 0.23, *P* >0.05) *mRNAs*.

### Mesodermal-specific *Alk5* inactivation reduces the sub-mesothelial mesenchyme

**Fig. 1:**
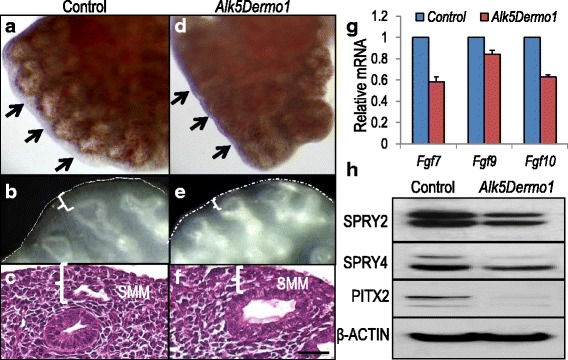
Mesodermal progenitor-specific deletion of *Alk5* reduced the sub-mesothelial mesenchyme. (**a**–**f**) Gross morphology (**a**, **d**, **b**, and **e**) and hematoxylin and eosin staining (**c** and **f**) show decreased sub-mesothelial mesenchyme in *Alk5* ^*Dermo1*^ mutant lungs at E12.5 (**b**, **e**, **c**, and **f**) or E13.5 (**a** and **d**). (**g**) Quantitative PCR showed decreased *Fgf* 7, *Fgf9*, and *Fgf10* mRNA in E13.5 *Alk5* ^*Dermo1*^ lungs; *n* = 2 controls and 3 mutant lungs. (**h**) Western blot analysis showed decreased FGF signaling target genes. β-ACTIN was used as control, *n* = 3. Error bars show standard deviation. Scale bar: f = 20 μm

Structurally, the SMM [15] of *Alk5*^*Dermo1*^ lungs was smaller and more irregularly shaped compared to control lungs (Fig. 1a–e and Additional file 4). Anti-phospho-histone H3 labeling revealed fewer mitotic cells within the distal mesenchyme of mutant lungs (2.02 % vs*.* 0.6 %). The SMM regulates epithelial, smooth muscle, and vascular development by producing factors such as FGF10 [18]. FGF10 mRNA in mutant lungs was 0.63 ± 0.02 of controls (Fig. 1g). In addition, *Fgf9* made by the mesothelium/epithelium and *Fgf7* expressed by the mesenchyme were respectively expressed at 0.84 ± 0.04 and 0.58 ± 0.05 of controls (Fig. 1g). To assess the functional significance of the reduced *Fgf10* and *Fgf9* mRNAs, we examined expression of their target genes. Immunoblotting revealed decreased SPRY2 and SPRY4 in *Alk5*^*Dermo1*^ lungs relative to controls, confirming functionally reduced FGF10 signaling (Fig. 1h). Similarly, there was profound decrease in the homeodomain transcription factor PITX2, a major target of FGF9 (Fig. 1h). These data suggest that mesodermal *Alk5* inactivation reduces SMM volume by decreasing overall functional FGF signaling.

### Inhibition of airway smooth muscle development

**Fig. 2:**
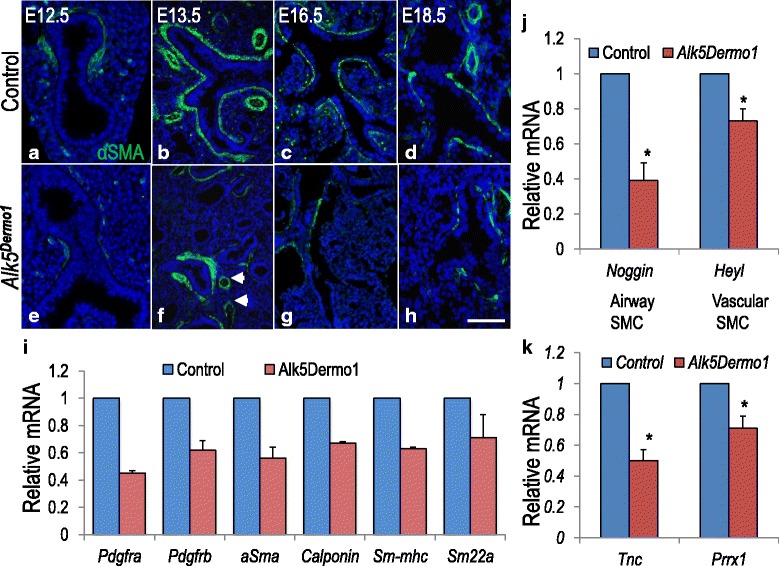
Mesodermal *Alk5* deficiency inhibits smooth muscle development. (**a**–**h**) Immunohistochemistry (IHC) showed αSMA is decreased throughout lung development in *Alk5* ^*Dermo1*^ lungs. (**i**) Quantitative PCR (Q-PCR) confirmed the repression of myofibroblast-related mRNAs in *Alk5* ^*Dermo1*^ lungs at E13.5. *n* = 2 pairs of separate lungs, repeated once. (**j**) Q-PCR analysis showed decreased *Noggin* with smaller loss in *Heyl* expression in *Alk5* ^*Dermo1*^ lungs at E13.5. *n* = 3 pairs of separate lungs. (**k**) Q-PCR showed decreased *Tnc* and *Prrx1* mRNA in *Alk5* ^*Dermo1*^ lungs. *n* = 3 pairs of separate lungs. Error bars show standard deviation. *P <0.05. Scale bar: h = 20 μm

**Fig. 3:**
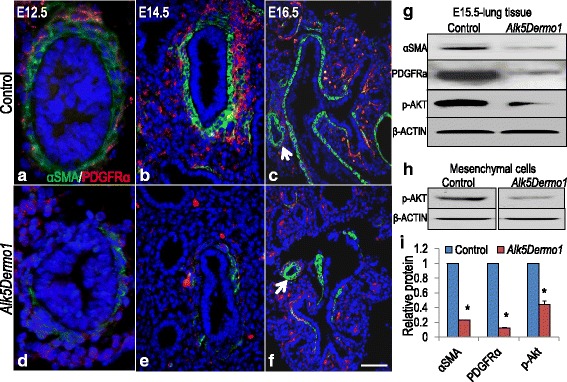
Mesodermal *Alk5* deficiency reduces smooth muscle precursors. (**a**–**f**) Immunohistochemistry showed reduced αSMA (green) and PDGFRα (red) in *Alk5* ^*Dermo1*^ lungs throughout lung development. (**g**) Western blot analysis showed decreased αSMA, PDGFRα and p-AKT protein in E15.5 *Alk5* ^*Dermo1*^ whole lung tissue. β-ACTIN was used as control. *n* = 3 separate lungs. (**h**) Western blot analysis showed decreased p-AKT protein in primary mesenchymal cells isolated from E15.5 *Alk5* ^*Dermo1*^ lungs. *n* = 3 pairs of separate lungs. β-ACTIN was used as control. (**i**) Densitometric analysis of western blot results in (**g**). Error bars show standard deviation. *P <0.05. Scale bar: f = 20 μm

FGF10^pos^ cells contribute to differentiated peribronchial smooth muscle (PBSM) cells [3]. By IHC, layers of αSMA^pos^ PBSM were thinner in diameter and included large gaps in E18.5 mutant lungs (Fig. 2a–h). However, αSMA expression around large blood vessels was similar in mutant and control lungs (Fig. 2f, Arrowheads). Consistent with the IHC, Q-PCR showed reduced mRNA for multiple myofibroblast markers in *Alk5*^*Dermo1*^ lungs (Fig. 2i). These included *αSMA* (0.56 ± 0.85), Calponin (0.67 ± 0.016), *SM-MHC* (0.63 ± 0.016), and *SM22α* (0.71 ± 0.18). In addition, there was reduced mRNA for both *Pdgfrα* (0.45 ± 0.021) and *Pdgfrβ* (0.625 ± 0.073). Transcripts for NOGGIN, an airway smooth muscle cell marker [19, 20], were also decreased (Fig. 2j, 0.39 ± 0.1 of controls, *P* <0.05) while those for *Heyl*, a vascular smooth muscle marker [20, 21], were only slightly reduced (Fig. 2j, 0.71 ± 0.078 of controls, *P* <0.05). Thus, the diminished αSMA^pos^ cells in *Alk5*^*Dermo1*^ lungs were primarily lost from the PBSM layers. In addition, there was decreased mRNA for paired-homeobox transcription factor PRRX1 (0.7 ± 0.056 of controls, *P <*0.05) and the extracellular matrix glycoprotein TENASCIN-C (TNC; 0.53 ± 0.047 of controls, *P* <0.01), both of which promote myofibroblast growth and differentiation (Fig. 2k). The effects of ALK5 abrogation on αSMA^pos^ cell commitment was also assessed by analysis of PDGFRα protein (Fig. 3a–f), which is regulated by TNC and highly expressed on αSMA^pos^ cell precursors [22]. The fraction of PDGFRα^pos^ cells surrounding the airway epithelium was significantly reduced in *Alk5*^*Dermo1*^ lungs (Fig. 3d–f), and western blot analysis confirmed the decrease in αSMA and PDGFRα (Fig. 3g, i). Consistent with this observation, phosphorylation of AKT, the canonical substrate of PDGFA-activated PDGFRα [23–25], was reduced in both *Alk5*^*Dermo1*^ lung homogenates (Fig. 3g) and isolated primary *Alk5*^*Dermo1*^ mesenchymal cells (Fig. 3h). Thus, ALK5-mediated TGFβ signaling is required in lung αSMA^pos^ cell development, and early defects in this pathway lead to decreased populations of cells expressing αSMA or PDGFRα.

### Lipofibroblast (LIF) hyperplasia in *Alk5*^*Dermo1*^ lungs

**Fig. 4:**
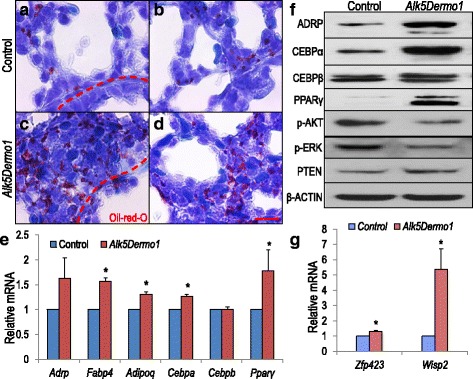
Lipofibroblast hyperplasia in *Alk5* ^*Dermo1*^ lungs. (**a**–**d**) Oil Red O-stained cells are increased in E18.5 *Alk5* ^*Dermo1*^ lungs. Dotted lines indicate basement membrane between epithelium and surrounding mesenchyme. (**e**) Quantitative PCR (Q-PCR) showed mRNA for LIF markers increased in E18.5 *Alk5* ^*Dermo1*^ lungs. *n* = 3 pairs of independent lungs, Error bars show standard deviation, *P <0.05. (**f**) Western blot analysis confirmed increased LIF markers, *n* = 6 pairs of lungs. Western blot analysis also showed decreased p-AKT and p-ERK, and increased PTEN. β-ACTIN was used as control. *n* = 3 pairs of lungs. (**g**) Q-PCR showed increased *Zfp423* and *Wisp2* mRNA *in Alk5* ^*Dermo1*^ lungs compared to controls. *n* = 5–6 pairs of independent lungs. Error bars show standard error of the mean. *P <0.05. Scale bar: d = 10 μm

In addition to αSMA^pos^ and endothelial cells, the multipotential lung mesoderm gives rise to LIFs. In the rat, LIFs are detected on embryonic day E16, increase during gestation, and decline after birth [26]. Oil Red O staining of *Alk5*^*Dermo1*^ lungs revealed overwhelmingly abundant LIFs within the developing mesoderm, relative to controls (Fig. 4a–d and Additional file 5). To confirm that observation, Q-PCR was used to quantify the mRNA for multiple LIF markers, including adipocyte transcription factors PPAR*γ* and CEBP*α*, the lipid droplet-associated Adipose Differentiation-Related Protein (ADRP), and adipocyte Fatty Acid Binding Protein 4 (FABP4). All were elevated in *Alk5*^*Dermo1*^ lungs (Fig. 4e). Immunoblots of total *Alk5*^*Dermo1*^ lung homogenates confirmed that ADRP, CEBPα, and PPARγ proteins were more abundant compared to controls. Furthermore, p-AKT and p-ERK were decreased while PTEN, a TGFβ-repressed phosphatase associated with LIF hyperplasia [27], was increased in *Alk5*^*Dermo1*^ lungs (Fig. 4f). Importantly, *Zfp423*, which encodes a zinc finger transcription factor that regulates PPAR*γ* in pre-adipocytes [28, 29], as well as WISP2, a matricellular protein that is highly expressed in adipocyte precursors [30, 31], were increased in *Alk5*^*Dermo1*^ lungs (Fig. 4g). These findings indicate that abrogation of ALK5-mediated signaling increases LIF commitment and differentiation.

### Establishment of *Alk5*-deficient mesodermal cell lines

**Fig. 5:**
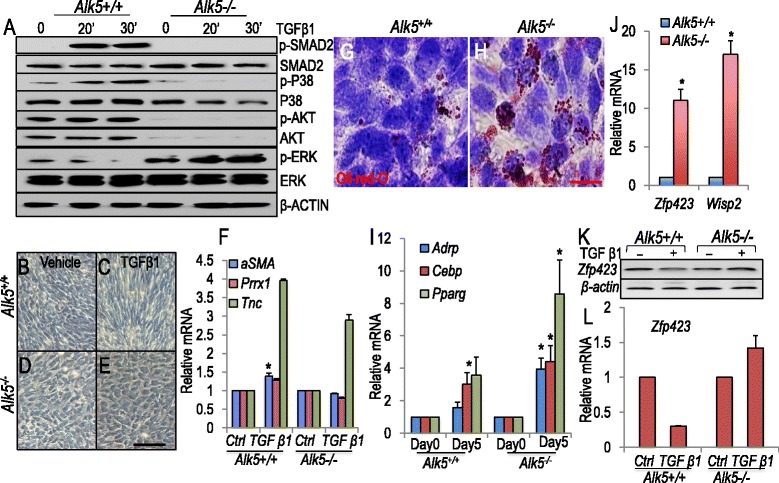
Reduced myogenesis and increased adipogenesis in SV40-trasformed, GFP-sorted *Alk5* ^*–/–*^ *cells*. (**a**) Western blot analysis of major signaling pathway components in SV40-trasformed and GFP sorted *Alk5* ^*+/+*^ and *Alk5* ^*–/–*^ cells treated with or without TGFβ1, *n* = 3 cell isolations from 6 pairs of lungs were used for each condition. (**b**–**e**) Representative images of *Alk5* ^*+/+*^ and *Alk5* ^*–/–*^ cells treated with (c and e) or without (b and d) TGFβ1 for 48 hrs. Note the morphology difference between *Alk5* ^*+/+*^ and *Alk5* ^*–/–*^ treated with TGFβ1 (**c** vs. **e**). (**f**) Quantitative PCR (Q-PCR) of mRNA isolated immediately after 48 hours of TGFβ treatment showed induced *αSMA* and *Prrx1* mRNA by TGFβ1 only in *Alk5* ^*+/+*^ cells, while *Tnc* mRNA was induced in both *Alk5* ^*+/+*^ and *Alk5* ^*–/–*^ cells. *n* = 2–3 cell isolations from 6 pairs of lungs per experiment, repeated once for each. (**g** and **h**) Oil Red O staining showed lipofibroblasts (LIFs) increased extensively in *Alk5* ^*–/–*^ mesenchymal cells, cultured in 2 % low serum for 5 days. (**i**) Q-PCR showed increased LIF-related gene expression in *Alk5* ^*–/–*^ cells cultured for 5 days. *n* = 4 cell isolations from 6 pairs of lungs per experiment, repeated 2 to 3 times. (**j**) Q-PCR showed increased *Zfp423* and *Wisp2* mRNA in *Alk5* ^*–/–*^ cells. *n* = 5 to 6 cell isolations from 6 pairs of lungs per experiment, repeated 1 to 4 times. **k**. Gel image of Q-PCR products quantified in (l) of *Zfp423* mRNA in *Alk5* ^*+/+*^ and *Alk5* ^*–/–*^ cells, treated with or without TGFβ1 for 48 hrs. *Zfp423* mRNA was decreased only in the *Alk5* ^*+/+*^ cells, treated with TGFβ1. *n* = 2 cell isolations from 6 pairs of lungs per experiment, repeated once for each. Error bars show standard error of the mean except for (**l**) which shows standard deviation. *P <0.01. Scale bars: e = 20 μm, h = 10 μm

The aggregate data presented above suggests that inactivation of *Alk5* in multipotential lung mesoderm promotes LIF commitment and inhibits SM differentiation. To examine the underlying mechanisms, we isolated mesodermal cells from *Alk5*^*Dermo1*^*;mTmG* and *Alk5*^*flox/flox*^*;mTmG* mouse lungs. These *Alk5*^*–/–*^ and *Alk5*^*+/+*^cells were immortalized by transfection with an SV40 plasmid and purified by FACS (Methods). TGFβ-induced SMAD2 phosphorylation was nearly absent in *Alk5*^*–/–*^ cells, confirming abrogation of ALK5-mediated canonical TGFβ signaling (Fig. 5a). Other mediators of TGFβ signaling were also affected. Baseline p-P38, p-AKT and p-ERK were high in *Alk5*^*+/+*^ control cells but TGFβ stimulation minimally induced p38 activation in mutant cells. TGFβ also had little impact on p-AKT in the mutant cells, but total AKT was reduced. Importantly, baseline ERK phosphorylation was elevated in *Alk5*^*–/–*^ cells, and TGFβ induced a robust response in these cells compared to *Alk5*^*+/+*^ controls (Fig. 5a). These findings indicate the critical and complex role of ALK5 activity in the TGFβ signal transduction pathway.

### ALK5-mediated TGFβ represses the LIF transcription factor *ZFP423* in vitro

We also evaluated the impact of TGFβ on the isolated cells. Treatment with recombinant TGFβ (4 ng/mL for 48 hours, Materials & Methods) initiated myogenic differentiation as assessed by increased *αSMA* and *Prrx1* transcripts in *Alk5*^*+/+*^ but not *Alk5*^*–/–*^ mesenchymal cells (Fig. 5b–f). In contrast, *Tnc* induction by TGFβ was unaffected by *Alk5* inactivation, suggesting that its regulation is independent of ALK5 and the canonical TGFβ pathway. Conversely, *Alk5*^*–/–*^ cells displayed a significantly high rate of spontaneous adipogenic differentiation in non-induced, minimum standard culture medium (Fig. 5g, h). This did not occur in *Alk5*^*+/+*^ controls, and was verified by assessing the expression of adipogenic markers. After 5 days in culture, lipogenic mediators, including *Adrp*, *Cebpα* and *Pparγ* mRNA, increased by 1.57 ± 0.34-, 3.02 ± 0.7-, and 3.57 ± 1.1-fold, respectively in the *Alk5*^*–/–*^ cells compared to 3.95 ± 0.68-, 4.4 ± 1.0-, and 8.58 ± 2.1-fold in the *Alk5*^*+/+*^cells (Fig. 5i). Likewise, *Zfp423* mRNA was 11-fold higher and *Wisp2* mRNA was 17-fold higher in *Alk5*^*–/–*^cells relative to *Alk5*^*+/+*^ cells *(*Fig. 5j). To determine the relationship between TGFβ and *Zfp423*, we treated *Alk5*^*+/+*^ and *Alk5*^*–/–*^ cells with recombinant TGFβ ligand (4 ng/mL for 48 hours, Materials & Methods). Controls were treated exactly the same with the exception of bovine serum albumin (BSA) replacing TGFβ. TGFβ repressed *Zfp423* mRNA levels and this activity was dependent on ALK5 (Fig. 5k, l). Taken together, these data indicate a novel mechanism by which abrogation of ALK5*-*mediated canonical TGFβ signaling, through regulation of *Zfp423*, promotes LIF versus αSMA^pos^ cell differentiation in lung mesodermal cells.

### The role of PDGFRα

**Fig. 6:**
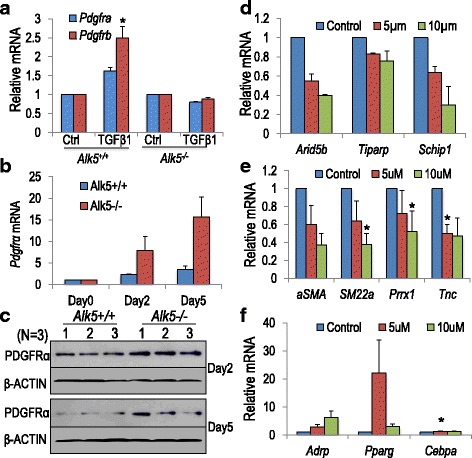
Role of *Pdgfrα*. (**a**) Quantitative PCR (Q-PCR) analysis showed induction (fold increase over controls) of *Pdgfrα* and *Pdgfrβ* mRNAs in response to 48 hours of TGFβ1 treatment only in *Alk5* ^*+/+*^ cells. Controls were treated exactly the same with the exception of bovine serum albumin in place of TGFβ. For each condition, the RNA was isolated from combined triplicate wells. For *Pdgfrα*, *n* = 2 cell isolations from 6 pairs of lungs per experiment, repeated 2 to 3 times. For *Pdgfrβ*, *n* = 3 cell isolations from 6 pairs of lungs per experiment. All conditions were initiated and analyzed after the same incubation period. (**b**) Progressive increase in *Pdgfrα* and *Pdgfrβ* mRNAs, without TGFβ1-induction during spontaneous differentiation of *Alk5* cell lines in culture for 0, 2, and 5 days. For each condition, the RNAs were isolated from combined triplicate wells. *n* = 2 cell isolations from 6 pairs of lungs per experiment, repeated once for each. In both (**a**) and (**b**), RNA was isolated at the indicated time point and Q-PCR was performed immediately after. Note higher levels in *Alk5* ^*–/–*^ compared to *Alk5* ^*+/+*^ cells on both day 2 and day 5. (**c**) Western blot analysis confirmed higher PDGFRα protein in three independent samples of *Alk5* ^*–/–*^ cells on both day 2 and day 5, compared to *Alk5* ^*+/+*^. *n* = 3 independent experiments. β-ACTIN was used as a control. (**d**–**f**) *Alk5* ^*+/+*^ cells were treated with or without Imatinib at 5 μm and 10 μm for 5 days, mRNA for PDGF signaling target genes (**d**), myofibroblast-related genes (**e**) and lipofibroblast-related genes (**f**) were analyzed by Q-PCR (*n* = 2 cell isolations from 6 pairs of lungs per experiment for (**d**) and *n* = 3 cell isolations from 6 pairs of lungs per experiment for (**e**) and (**f**). Error bars show standard deviation in (**d**) and standard error of the means in (**e**) and (**f**). *P <0.05

As noted above, PDGFRα and signaling were decreased in *Alk5*^*Dermo1*^ lungs (Fig. 3). Although PDGFRα^pos^ cells can differentiate into either αSMA^pos^ cells or LIFs, their role in determining mesodermal commitment has not been elucidated. We therefore sought to determine whether PDGFA signaling through PDGFRα is necessary for *Alk5* to induce αSMA^pos^ cell differentiation. TGFβ is known to induce *Pdgfrβ*; however, its role in regulating *Pdgfrα* remains unknown. Because PDGFRα is decreased in *Alk5*^*Dermo1*^ mutant lungs, its possible regulation by TGFβ was evaluated by TGFβ treatment (4 ng/mL, Materials & Methods) for 48 hours. Controls were treated exactly the same with the exception of BSA replacing TGFβ. TGFβ increased both *Pdgfrα* and *Pdgfrβ* mRNAs in *Alk5*^*+/+*^ cells but not in *Alk5*^*–/–*^ cells (Fig. 6a) indicating that TGFβ regulation of *Pdgfrα* and, hence, αSMA^pos^ cell differentiation, requires ALK5 activity. Surprisingly, *Pdgfrα* mRNA increased during spontaneous differentiation of *Alk5*^*–/–*^ cells to LIFs over a 5 day period (Fig. 6b). In addition, PDGFRα protein was consistently higher in *Alk5*^*–/–*^ compared to *Alk5*^*+/+*^ cells on both day 2 and day 5 of culture (Fig. 6c). These observations suggest the possibility of alternative or additional mechanisms that may regulate PDGFRα expression in these cells.

The need for signaling through PDGFRα during αSMA^pos^ cell differentiation was examined in vitro and in vivo. Application of the kinase inhibitor Imatinib to *Alk5*^*+/+*^ cells reduced the mRNA for known PDGFA targets *Arid5b*, *Tiparp*, and *Schip1* (Fig. 6d), thereby validating its use. Importantly, Imatinib reduced the expression of myogenic markers (Fig. 6e) but, of the LIF markers, only *Pparγ* was strongly increased. *Adrp* was modestly increased by higher Imatinib doses (Fig. 6f). These results suggest that *Pdgfra* inactivation in mesodermal precursors prevents αSMA^pos^ cell differentiation but is insufficient to promote LIF commitment.

**Fig. 7:**
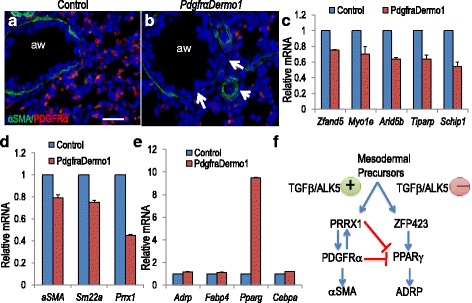
Mesodermal progenitor-specific deletion of *Pdgfra*. (**a** and **b**) Immunohistochemistry showed PDGFRα (red) is lost in the lung mesenchyme and αSMA (green) is decreased in E18.5 *Pdgfra* ^*Dermo1*^ lungs. Arrows indicate gaps in αSMA in mutant lungs. “aw” indicates airway. (**c**) Quantitative PCR (Q-PCR) showed a decrease in mRNA for PDGF signaling target genes, indicating an overall functional repression of the signaling pathway in E18.5 *Pdgfra* ^*Dermo1*^ lungs. *n* = 2 pairs of separate lungs, repeated once for each. (**d**) Q-PCR showed myofibroblast-related genes are decreased in E18.5 *Pdgfra* ^*Dermo1*^ lungs. *n* = 2 pairs of separate lungs, repeated once. (**e**) Q-PCR showing only *Pparγ* is robustly increased among lipofibroblast-related genes in E18.5 *Pdgfra* ^*Dermo1*^ lungs. *n* = 2 pairs of separate lungs, repeated once. (**f**) Schematic model for ALK5 binary switch function in mesodermal progenitor cell fate determination. TGFβ via ALK5 is a direct activator of *Prrx1* (Fig. 5f) a key transcriptional regulator of the αSMA^pos^ cell pathway. TGFβ via ALK5 also directly stimulates *Pdgfrα* mRNA (Fig. 6a), which further promotes αSMA^pos^ cell commitment [22]. PRRX1 and PDGFRα are reciprocal activators. In the absence of Alk5, *Zfp423*, which is normally repressed by TGFβ (Fig. 5l) is de-repressed, leading to activation of *Pparγ* [28], a transcriptional activator of lipogenic cell commitment. PRRX1 and PDGFRα inhibit adipogenesis by directly repressing PPARγ [44]. Error bars show standard error of the mean. Scale bar: b = 20 μm

To confirm this finding in vivo, genetically engineered mice were generated in which *Pdgfrα* was inactivated in mesodermal tissues by *Dermo1-cre*. The resulting *Pdgfrα*^*flox/flox*^*;Dermo1-cre* mice (*Pdgfrα*^*Dermo1*^) survived to birth at predicted Mendelian ratios but were 22 % smaller than control littermates (1.45 ± 0.03 g vs. 1.14 ± 0.04 g, *P* <0.001). *Pdgfrα*^*Dermo1*^ mutant mice also developed spina bifida as reported in mice deficient in PDGFRα-stimulated phosphatidyl-inositol 3’ kinase activity [23]. IHC on E18.5 control lungs localized PDGFRα-expressing cells to the stroma and to areas surrounding the airways and blood vessels (Fig. 7a). These cells were absent or significantly reduced in *Pdgfra*^*Dermo1*^ lungs (Fig. 7b and Additional file 6). Q-PCR showed reduced mRNA for PDGFA targets, including *Zfand5*, *Myo1e*, *Arid5b*, *Tiparp*, and *Schip1* [32], confirming functionally repressed PDGFA signaling in *Pdgfrα*^*Dermo1*^ lungs (Fig. 7c and Additional file 7A). The αSMA^pos^ cell layers in mutant lungs were reproducibly thinner compared to controls, with gaps in αSMA expression (Fig. 7b, Arrows and Additional file 6). Q-PCR of E18.5 *Pdgfra*^*Dermo1*^ lungs showed decreased mRNA for multiple myofibroblast markers, including *Prrx1* (0.45 ± 0.01 of control), *SM22α* (0.75 ± 0.02), and *αSMA* (0.79 ± 0.03) (Fig. 7d). Myofibroblast marker mRNAs were similarly decreased in E14.5 *Pdgfra*^*Dermo1*^ lungs (Additional file 7B). Similar analysis of adipogenic-related genes in *Pdgfra*^*Dermo1*^ lungs at E18.5 showed increased *Pparγ* mRNA but only modest increases in *Adrp*, *Fabp4*, and *Cebpα* (Fig. 7e). The balance between αSMA^pos^ cells and LIF phenotypes in *Pdgfra*^*Dermo1*^ lungs was less severely distorted than those in *Alk5*^*Dermo1*^ mice. In sum, these data support a model whereby ALK5-mediated TGFβ signaling regulates αSMA^pos^ versus LIF cell differentiation only partly through the PDGFA/PDGFRα pathway and that inhibition of this pathway is insufficient to promote LIF differentiation.

## Discussion and conclusions

The present study provides genetic, histologic, and molecular evidence that mesodermal inactivation of *Alk5* in the mouse lung restricts αSMA^pos^ cell fate and promotes LIF differentiation. In this context, ALK5 meets the definition of a molecular binary switch that functions in determination and subsequent specialization of at least two key lung mesodermal cell lineages.

An early morphological phenotype of *Alk5*^*Dermo1*^ embryonic lungs is the reduced physical size of the SMM. This was associated with decreased proliferation of the lung mesoderm and reduction in *Fgf9. Fgf9* is expressed by both the mesothelium and the epithelial layers in the lung [1, 2]. *Dermo1-cre* is not known to be active in the mesothelium, although the origin of some mesothelial cells may be traced to the pulmonary mesoderm [33]. Whether reduced *Fgf9* is due to direct impact of *Alk5* inactivation or a result of mesoderm-mesothelial cross communication remains unknown. FGF9 signaling through FGFR1 and FGFR2 is both necessary and sufficient for SMM growth. Although *Alk5* inactivation modestly decreased *Fgf9*, PITX2, a homeodomain transcription factor that is induced by FGF9, was strongly inhibited (Fig. 1k). SMM volume is profoundly sensitive to FGF9 signaling, being entirely absent from *Fgf9*^*–/–*^ lungs and enlarged in *Fgf9* gain-of-function mutants [1]. The reduced SMM size and mitotic index indicate that ALK5 is required for either mesothelial *Fgf9* expression or FGF9 responsiveness. Ligand-dependent TβRII signaling has been shown to regulate FGF9- and PITX2-mediated cell proliferation in the palatal mesenchyme [34]. Our observations suggest that ALK5 may similarly mediate TGFβ-mediated control of FGF9 signaling within the SMM.

The *Alk5*^*Dermo1*^ lungs also displayed defective epithelial differentiation. As *Dermo1* is exclusively mesodermal, the abnormalities in epithelial cell differentiation presumably represent altered mesenchymal regulation of epithelial progenitors. Previous studies have suggested that FGF7, which is reduced in *Alk5*^*Dermo1*^ lungs, participates in mesenchymal-epithelial cross-talk [2]. Alternatively, TGFβ also regulates epithelial differentiation by altering the composition of the underlying matrix [35]. Finally, TUNEL^pos^ cells were found scattered in the parenchyma of late stage (i.e. E18.5) *Alk5*^*Dermo1*^ lungs (Additional file 8). Although these cells are not localized to the airways, the possibility that some of the changes in airway gene expression may be due to increased apoptosis cannot be presently ruled out.

The loss of *Alk5* in mesodermal progenitors also disrupted pulmonary vasculogenesis. *Flk1*, also known as vascular endothelial growth factor receptor 2, is expressed by endothelial progenitors [36] and was strongly decreased in E13.5 *Alk5*^*Dermo1*^ lungs. This likely accounts for the simplified vasculature observed in E18.5 mutant lungs. Since *Dermo1* may not be expressed in endothelial progenitors [37, 38], it is unclear whether *Alk5* directly participates in vascular endothelial cell commitment. In addition, reduced *Fgf9* in *Alk5*^*Dermo1*^ lungs might also contribute to the observed vascular phenotype. Increased *Fgf9* in vivo is sufficient to expand the network of *Tie2-lacZ* and PECAM^pos^ cells through stimulation of undifferentiated mesenchyme and/or vascular progenitor cells [1].

The key finding of the present study is that inactivation of *Alk5* inhibits αSMA^pos^ and promotes LIF differentiation, both in vivo and in cultured lung mesodermal cell lines. Markers of myofibroblast differentiation, including αSMA, SM22α, and calponin, were reduced, while mediators of lipogenic commitment, such as ADRP, were robustly increased. The mechanism underlying this phenotype is complex. Since TGFβ signaling is known to induce αSMA^pos^ cell differentiation, its inhibition is readily explicable by ALK5 deficiency. In addition, FGF10^pos^ cells are known to contribute to αSMA^pos^ cells during lung development [3]. FGF10 mRNA was reduced as was the physical size of SMM, the site of FGF10^pos^ cells. In contrast to this finding, we had previously found increased *Fgf10* mRNA and SHH signaling in mesodermally-targeted *TβR2*^*Dermo1*^ lungs [39]. The discrepancy between the previous and present observations on FGF10 provides an excellent illustration of differential TGFβ signaling via receptor selectivity as described in the Background section. Thus, receptor selectivity (TβR2 vs. ALK5) represents an important mechanism by which TGFβ can impose a vast spectrum of physiologic changes on different (or same) tissues and cell types. In the present study, when the observation on reduced *Fgf10* levels in the *Alk5*^*Dermo1*^ lungs is combined with a diminished mitotic cell index in the SMM compartment, the data suggest a decreased number of FGF10^pos^ cells and hence reduced αSMA^pos^ cell differentiation. Consistent with this conclusion, histological analysis of *Alk5*^*Dermo1*^ lungs revealed reduced αSMA^pos^ cell differentiation in the PBSM but not in perivascular smooth muscle cells (Fig. 2f). It is unclear whether PBSM cells and vascular smooth muscle cells have common or distinct developmental origins.

PDGFRα is also expressed by αSMA^pos^ progenitors and was similarly and profoundly reduced in *Alk5*^*Dermo1*^ lungs. Its functional abrogation was confirmed by reduced phosphorylation of AKT, its principle intracellular substrate (Fig. 3). Our study showed that the TGFβ-ALK5 axis regulates *Pdgfrα* mRNA expression (Fig. 6a). Therefore, it was surprising to find that *Pdgfrα* mRNA increased during spontaneous differentiation of *Alk5*^*–/–*^ cells to LIFs (Fig. 6b). It is likely that in these isolated cells alternative or additional mechanisms may exist which regulate *Pdgfrα* expression, independent of the TGFβ-ALK5 axis. One potential mechanism may be increased spontaneous phosphorylation of ERK found in *Alk5*^*–/–*^ cells compared to *Alk5*^*+/+*^cells and *Alk5*^*Dermo1*^ lungs (Figs. 4 and 5). The underlying cause of increased p-ERK in cells lacking ALK5 activity is outside the scope of the present study, but is being currently investigated in separate studies. What remains firmly established from these in vitro studies is that inhibition of PDGFRα by Imatinib reduced expression of myofibroblast markers but did not fully promote LIF differentiation; of the lipogenic genes, only *Pparγ* was increased. This finding was validated by genetic targeting of *Pdgfrα* in vivo (Fig. 7). Inactivation of *Pdgfrα* in early mesodermal progenitors (*Pdgfra*^*Dermo1*^) robustly inhibited the expression of myofibroblast-specific genes (Fig. 7d), while, compared to *Alk5*^*Dermo1*^ lungs, the impact on LIF differentiation was subtle, despite strong activation of *Pparγ* in cultured cells. Thus, while PDGFRα is required for full expression of the myofibroblast-specific gene battery, its absence is capable of initiating, but not completing, the LIF differentiation program.

LIFs are critical regulators of alveologenesis and assimilate and transfer neutral lipids to adjacent type II cells, thereby facilitating surfactant formation [40]. LIF commitment may be favored by increased expression of PTEN, a phosphatase that regulates the fate of mesodermal progenitors (Fig. 4f). This finding recapitulates our previous study in which *Dermo1-cre*-mediated inactivation of *Pten* profoundly reduced ADRP [14]. *Pten* was originally identified as a negatively regulated target of TGFβ signaling [41].

Based on the collective observations in this study we propose a model whereby multiple signaling pathways, controlled by ALK5-mediated TGFβ signaling, converge on transcriptional factors that regulate the cell lineage determination of two key mesodermal specialized cell types (Fig. 7f). The two major transcription factor targets of this process include the adipogenic zinc finger transcription factor ZFP423, and the myogenic transcription factor PRRX1. Activation of *Zfp423* through knockdown of *Zfp521*, its negative regulator, is capable of altering mesodermal cell fate from osteogenic to lipogenic lineage [42]. We found increased LIFs in *Alk5*^*Dermo1*^ lungs associated with increased *Zfp423* mRNA. Likewise, *Zfp423* increased robustly in cultured *Alk5*^*–/–*^ cells undergoing spontaneous LIF differentiation (Fig. 5j). Importantly, TGFβ1 strongly repressed *Zfp423*, and only in presence of functional ALK5 (Fig. 5l). Thus, while ALK5 activity is required for αSMA^pos^ cell lineage commitment, its inactivation de-represses *Zfp423* favoring early LIF lineage commitment. In support of this conclusion, we found profoundly increased expression of *Wisp2*, known to be expressed at high levels in committed adipocyte precursors. In contrast, PRRX1 is a mesodermal transcription factor [43] that promotes differentiation of mesenchymal precursors into αSMA^pos^ cells. PRRX1 inhibits adipogenesis while its stable knockdown enhances adipogenesis, as shown by increased *Pparγ*, *Cebpα*, and *Fabp4* expression [44]. In *Alk5*^*Dermo1*^ lungs, *Prrx1* decreased as *Zfp423* increased, indicating a key mechanism by which *Alk5* repression facilitates LIF commitment while inhibiting αSMA^pos^ cell differentiation.

In conclusion, these data establish that ALK5 has a regulatory role in the control of early mesodermal progenitor commitment between two alternative cell lineages. This role is consistent with previous in vitro studies wherein genome-wide pathway analysis identified members of the TGFβ superfamily as modulators of adipocyte/myocyte differentiation [45]. αSMA^pos^ cell determination is regulated by TGFβ signaling and requires further signaling by FGF9 and PDGFRα. In the wild type multipotential mesoderm, activation of the αSMA^pos^ cell pathway, mediated by the transcription factor PRRX1 also limits the ontogeny of LIFs. Blocking ALK5-mediated TGFβ signaling in *ALK5*^*Dermo1*^ mesoderm inhibited αSMA^pos^ cell differentiation and increased (de-repressed) the expression of *Zfp423*, the key LIF transcription factor [28]. Therefore, it appears that, while αSMA^pos^ cell lineage is actively determined by ALK5-mediated TGFβ signaling, LIF determination represents a default position on a biological binary switch. This means that LIF can be activated only when αSMA^pos^-promoting stimuli are diminished or eliminated.

## Methods

### Animals

The *Alk5*^*flox/flox*^ (C57BL6 background) and *Dermo1-cre* mice were described previously [39, 46]. *Pdgfra*^*flox/flox*^ mice were purchased from the Jackson Laboratory. Mesodermal progenitor specific knockout mutants, *Alk5*^*Dermo1*^ (*Alk5*^*flox/flox*^*;Dermo1-cre*) or *Pdgfra*^*Dermo1*^ (*Pdgfra*^*flox/flox*^*;Dermo1-cre*) were generated by crossing *Alk5*^*flox/flox*^ or *Pdgfra*^*flox/flox*^ homozygous females with *Alk5*^*flox/+*^*;Dermo1-cre* or *Pdgfra*^*flox/+*^*;Dermo1-cre* double heterozygous males in a pure C57BL6 background. *Alk5*^*flox/flox*^ or *Pdgfra*^*flox/flox*^ mice were used as control. Triple transgenic *Alk5*^*flox/flox*^*;Dermo1-cre;mTmG* mice (simply *Alk5*^*Dermo1*^*;mTmG*) were generated as previously reported [47]. Genotyping of the *Dermo1-cre* mice containing *Alk5*^*flox*^, *Alk5*^*Δ*^, and *Alk5*^*wt*^ alleles was as previously described [39, 46]. All animal procedures were performed according to approved University of Southern California Institutional Animal Care and Use Committee regulations.

### Tissue collection and Oil Red O staining

Embryonic lungs from control and mutant lungs were collected at various embryonic stages from E11.5 to E18.5. The embryos or lungs were dissected and fixed overnight in 4 % paraformaldehyde at 4 °C. They were then dehydrated through increasing ethanol concentration and embedded in paraffin or OCT compound. Sections of 5-μm thickness were used for histological analysis. Frozen lung sections or cultured cells were stained with Oil Red O to detect LIFs according to the manufacturer’s protocol (Poly Scientific, Bayshore, NY, USA). The experiments were repeated using three mice within each genotype group (*n* = 3). For raw data pertaining to quantification of Oil Red O positive cells in Additional file 5, please see Additional file 9.

### TUNEL assay

Apoptotic cells were detected by using TUNEL detection kit (In Situ Cell Death Detection Kit, Roche Diagnostics, Indianapolis, IN, USA). Briefly, tissue sections were deparaffinized, rehydrated, and washed with distilled-deionized water. After treatment with proteinase K (Invitrogen, Carlsbad, CA, USA), fragmented DNA was labeled with fluorescein-dUTP, using terminal transferase (Roche Diagnostics). Slides were mounted with VECTASHIELD containing DAPI (Vector Laboratories, Burlingame, CA, USA). The apoptotic percentage was obtained as previously described [12] by manual counting of TUNEL^pos^ cells in groups of 4,000 or more cells.

### Immunohistochemistry (IHC)

Routinely prepared histological sections were deparaffinized with xylene and rehydrated through an alcohol gradient series to water. Antigens were retrieved and endogenous peroxidase activity was quenched using 3 % hydrogen peroxide. After normal serum blocking, the sections were incubated with a primary antibody at 4 °C overnight. Impress-anti-rabbit or anti-mouse or anti-goat IgG (Vector Laboratories) were applied for 50 min at room temperature. Staining was visualized by Peroxidase Substrate Kit DAB (Vector Laboratories). For immunofluorescence staining, the sections were incubated with primary antibodies overnight at 4 °C. After washing steps, the sections were reacted with a mixture of DyLight™549-conjugated donkey anti-rabbit or anti-mouse IgG (H + L) or DyLight™488-conjugated donkey anti-mouse IgG or anti-goat IgG (H + L) (all from Jackson ImmunoResearch Laboratories, Inc., West Grove, PA, USA) for 1 h in the dark at room temperature. After thorough rinses with PBS containing 0.1 % Triton X-100, the sections were mounted with VECTASHIELD mounting medium containing DAPI (to visualize nuclei). Primary antibodies used are described in Additional file 10. The experiments were repeated in more than three mice within each genotype group (n >3).

### Mesenchymal cell isolation, *SV40* transformation, Fluorescence Activated Cell Sorting (FACS) and treatment

Primary mesenchymal cells were isolated by differential adherence [48]. Briefly, fetuses were dissected from pregnant control and mutant mice at E15.5 or E16.5. Six pairs of lungs were pooled for each experiment (*n* = 6). Lungs were dissociated with 0.025 % trypsin/EDTA in DMEM (Gibco) at 37 °C. The cells were filtered and plated in 75 cm^2^ tissue culture flasks for 1 h, after which the non-adherent cells were discarded. Adherent cells were then washed and grown for 24 h. The primary cells were subsequently transformed by SV40 infection and subjected to FACS based on GFP activity using AriaII (Becton Dickinson). Gates were set according to unstained controls. After FACS, GFP^pos^ cells were either plated on 6-well plates in DMEM, containing 10 % fetal bovine serum, FBS (Gibco) for subsequent analysis, or directly subjected to Q-PCR.

For TGFβ1 (R&D Systems, Minneapolis, MN, USA) or Imatinib (Cayman Chemical Co. Ann Arbor, MI, USA) treatment, the purified *Alk5*^*+/+*^*or Alk5*^*–/–*^ cells were plated at a density of 5 × 10^4^ cells/cm^2^ into 6-well tissue culture plates in DMEM with 10 % FBS. Cells were serum-starved overnight before treatment with TGFβ1 (4 ng/mL according to the manufacturer’s recommendation) or imatinib (5 μL and 10 μL). TGFβ treatment was for 48 h; Imatinib treatment was for 5 days. Controls were treated exactly the same with the exception of BSA in place of TGFβ and DMSO in place of Imatinib. All conditions in Fig. 6a (Control, Alk5+/+, Alk5^–/–^) were initiated and analyzed after the same incubation period.

For adipogenesis differentiation, low-passage (<6 passages) *Alk5*^*+/+*^ and *Alk5*^*–/–*^ cells were plated at a density of 5 × 10^4^ cells/cm^2^ in DMEM containing 10 % FBS. At 80 % confluence, old medium was aspirated and replaced with fresh DMEM containing 2 % FBS. After 2, 5, or 10 days of incubation, the cells were stained with Oil Red O according to the manufacturer’s protocol (Poly Scientific, Bayshore, NY, USA) or prepared for RNA and protein analysis. *Alk5*^*+/+*^ and *Alk5*^*–/–*^ cells at 80 % confluence were used as a control. All experiments were performed in duplicate three times (*n* = 3).

### Western blot analysis

Total protein was extracted with RIPA buffer (Sigma, St. Louis, MO, USA) from embryonic lung tissues or cultured cells. Protein concentrations were determined by a BCA Protein Assay kit (Thermo Scientific, Grand Island, NY, USA); 15–30 μg of protein was loaded onto 3–8 % NuPAGE gels and transferred to Immobilon-P membranes (both from Millipore Corp, Billerica, MA, USA). Membranes were then blocked with 5 % milk in Tris-buffered saline and incubated with primary antibodies overnight. The secondary antibodies, goat-anti-rabbit or goat-anti-mouse IgG-HRP (both from Pierce, Thermo Scientific), were applied at a 1:1000 dilution for 30 min. Membranes were reacted with chemiluminescence reagent ECL (Amersham Biosciences, GE Healthcare Life Sciences, PA, USA) and exposed to photographic film (Amersham Hyperfilm ECL). The primary antibodies used are described in Additional file 10.

### Quantitative Polymerase Chain Reaction (Q-PCR)

DNase-free RNA was prepared using Trizol reagent (Invitrogen) according to the manufacturer’s instructions. After DNase treatment, total RNA (2 μg) was reverse-transcribed to cDNA using the Superscript III First-Strand Synthesis System for RT-PCR kit (Invitrogen), according to the manufacturer’s instructions. The cDNA was subjected to Q-PCR using SYBR Green PCR Master Mix with a LightCycler (Roche) as previously described [47]. Gene expression was normalized to *β-actin* or *Tbp*. All primers for Q-PCR were designed by using the program of Universal Probe Library Assay Design Center from Roche Applied Sciences. Specificity of each PCR reaction was verified by electrophoresis on 2 % gel.

Relative quantification analysis was carried out with LightCycler software, Version 4 (Roche). The results were expressed as a normalized ratio (fold). *β-actin* or *Tbp* were used as reference genes for normalization. Samples from control lungs served as ‘Calibrators’. For raw data pertaining to Q-PCR studies, please see Additional file 9.

### Statistical analysis

In total, the lungs or embryos from 98-pair *Alk5*^*Dermo1*^ mutant and control mice were analyzed using different techniques. For Q-PCR and western blot analysis, most experiments were performed two or three times, and the samples in each experiment were analyzed in triplicates or duplicates. All data are represented as mean ± standard error or standard error of the mean. The significance of differences between two sample means was determined by the two-tailed Student *t*-test, which is a common and well established statistical method to ascertain differences between two samples. Level of significance was denoted by *P* <0.05.

### Availability of supporting data

Data supporting the results of this article are available in Additional file 9.

## Additional files

Additional file 1:
*Dermo1-cre*-mediated mesodermal progenitor-specific deletion of *Alk5.* A–C. *Dermo1-cre* expression pattern in E14.5 *Dermo1-cre;mTmG* lungs. *Dermo1-cre*-mediated recombination (green fluorescence) was located in the mesenchyme surrounding the trachea (A), bronchi (B), bronchioles and blood vessels (C), not in the epithelial cells. D. Deletion of *Alk5* exon 3 by crossing *Alk5* ^*flox/flox*^ to *Dermo1-cre* mice. E. Deletion of *Alk5* was validated by PCR with genomic DNA. F. Western blot analysis using total protein from E16.5 control and *Alk5* ^*Dermo1*^ lungs. *n* = 3. β-ACTIN was used as a control. G–J. Immunohistochemistry for ALK5 (G and I) and PAI-1 (H and J) showed lost or decreased ALK5 and PAI-1 (Arrows in I and J) in *Alk5* ^*Dermo1*^ lungs. Scale bars: C, J = 20 μm. (PPTX 925 kb) Additional file 2: Mesodermal-specific *Alk5* inactivation causes pulmonary hypoplasia. A–D. Gross morphology of E16.5 control (A and B) and *Alk5* ^*Dermo1*^ (C and D) lungs. E–J. PAS (E, H, G and J) and H&E (F and I) staining of E18.5 control (E–G) and *Alk5* ^*Dermo1*^ (H–J) lungs. Arrows in I indicate a thicker alveolar wall in *Alk5* ^*Dermo1*^ lungs; Arrows in J indicate robust PAS staining in *Alk5* ^*Dermo1*^ lungs. Scale bars: H = 100 μm; I = 10 μm, J = 20 μm. (PPTX 1736 kb) Additional file 3: Mesodermal *Alk5* deficiency inhibits epithelial cell differentiation and disrupts vasculogenesis. A–H. Immunohistochemistry (IHC) for Acetylated-tubulin (A and E), CC10 (B and F), pro-SPC (C and G) and T1a (D and H) in E18.5 control and *Alk5* ^*Dermo1*^ lungs showing decreased expression of the latter genes in *Alk5* ^*Dermo1*^ lungs. I. Q-PCR confirmed inhibition of epithelial cell markers in *Alk5* ^*Dermo1*^ lungs, *n* = 2–9 pairs of separate lungs. J and L: IHC for GFP and FLK1 showing decreased GFP^+^/FLK1^+^ cells in E14.5 *Alk5* ^*Dermo1*^ lungs. K and M: IHC showed decreased CD34^+^ cells in E14.5 *Alk5* ^*Dermo1*^ lungs. N–Q. IHC showing decreased of PECAM1^+^ cells in E18.5 *Alk5* ^*Dermo1*^ lungs. Arrows indicate large blood vessels appearing intact. R. Quantitative PCR confirmed decreased *Pecam1*, *Flk1*, and *Flt1* mRNAs in E13.5 mutant lungs. *n* = 3 pairs of separate lungs. Error bars show standard deviation. **P* <0.05. Scale bars: H = 30 μm; M and Q = 20 μm. (PPTX 1560 kb) Additional file 4: Quantification of sub-mesothelial mesenchyme (SMM) size. Hematoxylin and eosin-stained lung tissue sections were prepared and random images were collected under 40× objective from both control and *Alk5* ^*Dermo1*^ embryos at E12.5 and E13.5. The various dimensions that were measured included boundaries of the SMM compartments, as delineated on each photomicrograph, from mesothelium to the mesenchyme wrapped around the epithelium (SEM). The regions encompassing the SMM were measured in Photoshop by dividing each region at random of six points to manually calculate the length from mesothelium to SEM (μm). Sample size, *n* = 3 separate lung tissue sections. Error bars show standard deviation. **P* <0.05. (PPTX 75 kb) Additional file 5: Quantification of Oil Red O storage. Lung tissue sections from E18.5 control and *Alk5* ^*Dermo1*^ lungs were analyzed by staining with Oil Red O for assessment of LIF differentiation. Oil Red O stained sections were viewed and random images were collected under 40× objective, and were analyzed by imaging using a previously described protocol [49], *n* = 3. The density of Oil Red O stained cells was then calculated and plotted as shown. Error bars show the standard deviation. **P* <0.05 (PPTX 43 kb) Additional file 6: Quantification of smooth muscle (SM) thickness and areas covered by smooth muscle in control and *Pdgfrα* ^*Dermo1*^ lungs at E18.5. αSMA immunohistochemical stained lung sections were collected by random sampling using the 40× objective from three pairs of control and *Pdgfrα* ^*Dermo1*^ mutant lungs at E18.5. The thickness and coverage of SM were quantified. A. Quantification of SM thickness. The arithmetic mean thickness of the SM cell layer was determined by volume of αSMA^pos^ compartment, measured by counting all points intercepting the airway epithelium and αSMA^pos^ compartment. *n* = 3. B. Quantification of SM coverage. The percentage of SM airway coverage was determined by assessing the percentage of the airway epithelium circumference in contact with number of αSMA^pos^ cells. *n* = 3. Error bars show the standard deviation. **P* <0.05 (PPTX 54 kb) Additional file 7: Mesodermal progenitor-specific deletion of *Pdgfrα.* A. Quantitative PCR (Q-PCR) showed PDGF signaling target genes are decreased, indicating an overall functional repression of PDGFA signaling pathway in E14.5 *Pdgfra* ^*Dermo1*^ lungs. *n* = 2 pairs of separate lungs, repeated twice or thrice. B. Q-PCR showed repression of myofibroblast-related genes in *Pdgfra* ^*Dermo1*^ lungs. *n* = 2 pairs of separate lungs, repeated thrice for each. Error bars show SEM. (PPTX 68 kb) Additional file 8: Mesodermal *Alk5* deficiency increased cell apoptosis. The number of TUNEL^pos^ cells as determined by previously described methods [12]. A–D. TUNEL assay showed increased number of TUNEL^pos^ cells in the *Alk5* ^*Dermo1*^ lungs at E18.5. E. Quantification of the relative number of TUNEL^pos^ cells (green) per 4,000 total cells. Error bars show standard deviation. **P* <0.05. (PPTX 794 kb) Additional file 9: Raw data. (XLSX 247 kb) Additional file 10: Primary antibodies used in western blots and immunohistochemistry. (DOC 37 kb)

## Abbreviations

FACS: Fluorescence Activated Cell Sorting
IHC: Immunohistochemistry
LIF: Lipofibroblasts
PBSM: Peribronchial smooth muscle
Q-PCR: Quantitative polymerase chain reaction
SM: Smooth muscle
SMM: Sub-mesothelial mesenchyme
TGFβ: Transforming growth factor beta
