# Supplementary material for: Mesodermal ALK5 controls lung myofibroblast versus lipofibroblast cell fate
Source: BMC Biol. 2016 Mar 16;14:19. doi: 10.1186/s12915-016-0242-9 (PMC4793501; doi:10.1186/s12915-016-0242-9)
Supplement: Additional file 1: — Dermo1-cre-mediated mesodermal progenitor-specific deletion of Alk5. A–C. Dermo1-cre expression pattern in E14.5 Dermo1-cre;mTmG lungs. Dermo1-cre-mediated recombination (green fluorescence) was located in the mesenchyme surrounding the trachea (A), bronchi (B), bronchioles and blood vessels (C), not in the epithelial cells. D. Deletion of Alk5 exon 3 by crossing Alk5 flox/flox to Dermo1-cre mice. E. Deletion of Alk5 was validated by PCR with genomic DNA. F. Western blot analysis using total protein from E16.5 control and Alk5 Dermo1 lungs. n = 3. β-ACTIN was used as a control. G–J. Immunohistochemistry for ALK5 (G and I) and PAI-1 (H and J) showed lost or decreased ALK5 and PAI-1 (Arrows in I and J) in Alk5 Dermo1 lungs. Scale bars: C, J = 20 μm. (PPTX 925 kb) [file 12915_2016_242_MOESM1_ESM.pptx]

## Slide 1
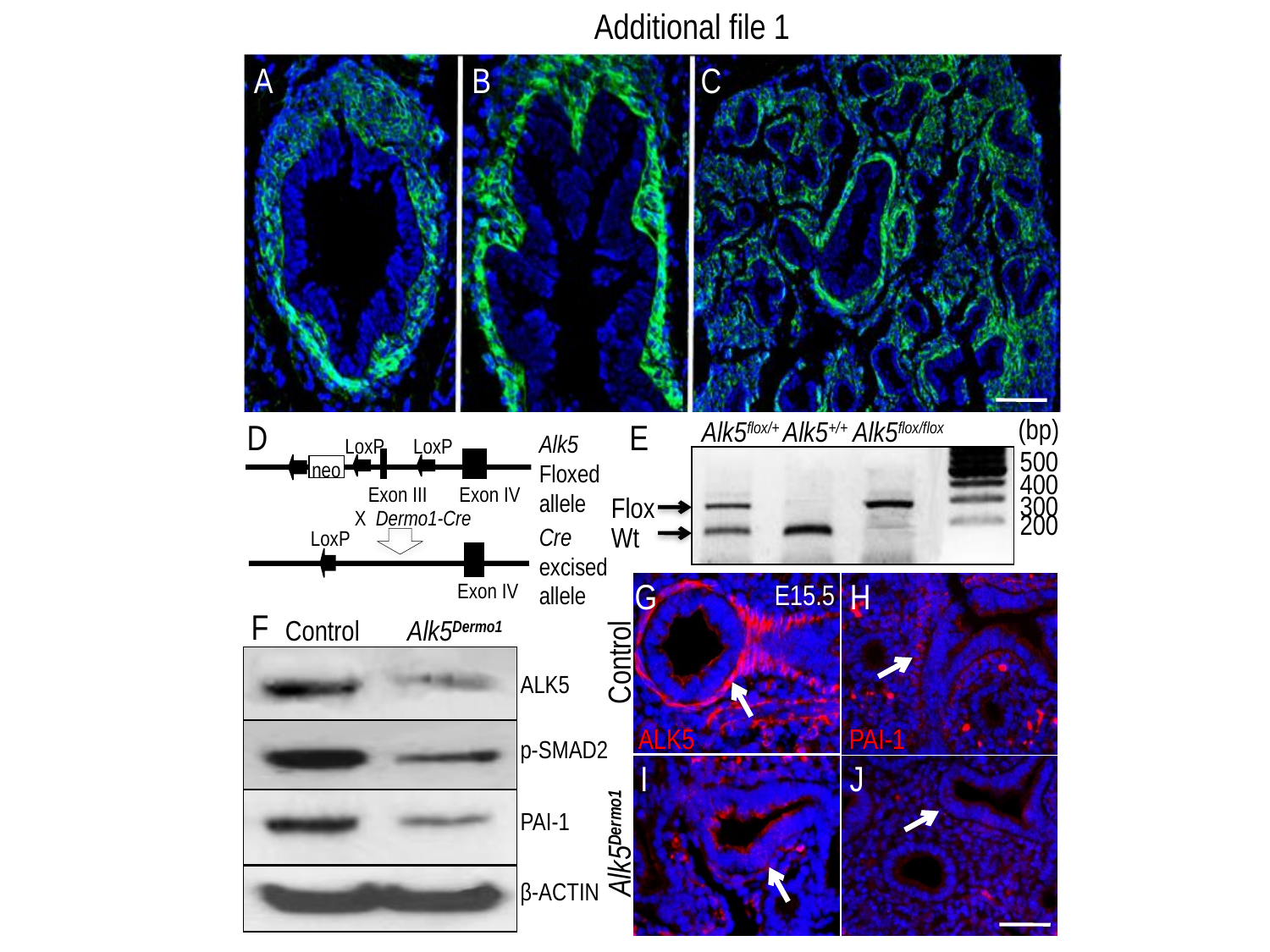

Additional file 1
A
B
C
(bp)
Alk5flox/+
Alk5+/+
Alk5flox/flox
500
400
300
Flox
200
Wt
E
D
Alk5
Floxed
allele
LoxP
LoxP
neo
Exon III
Exon IV
X Dermo1-Cre
Cre excised
allele
LoxP
Exon IV
G
H
E15.5
ALK5
PAI-1
I
J
F
Control
Alk5Dermo1
ALK5
p-SMAD2
PAI-1
β-ACTIN
Control
Alk5Dermo1
