# Supplementary figures and images for: Mesodermal ALK5 controls lung myofibroblast versus lipofibroblast cell fate
Source: BMC Biol. 2016 Mar 16;14:19. doi: 10.1186/s12915-016-0242-9 (PMC4793501; doi:10.1186/s12915-016-0242-9)

## Slide 1
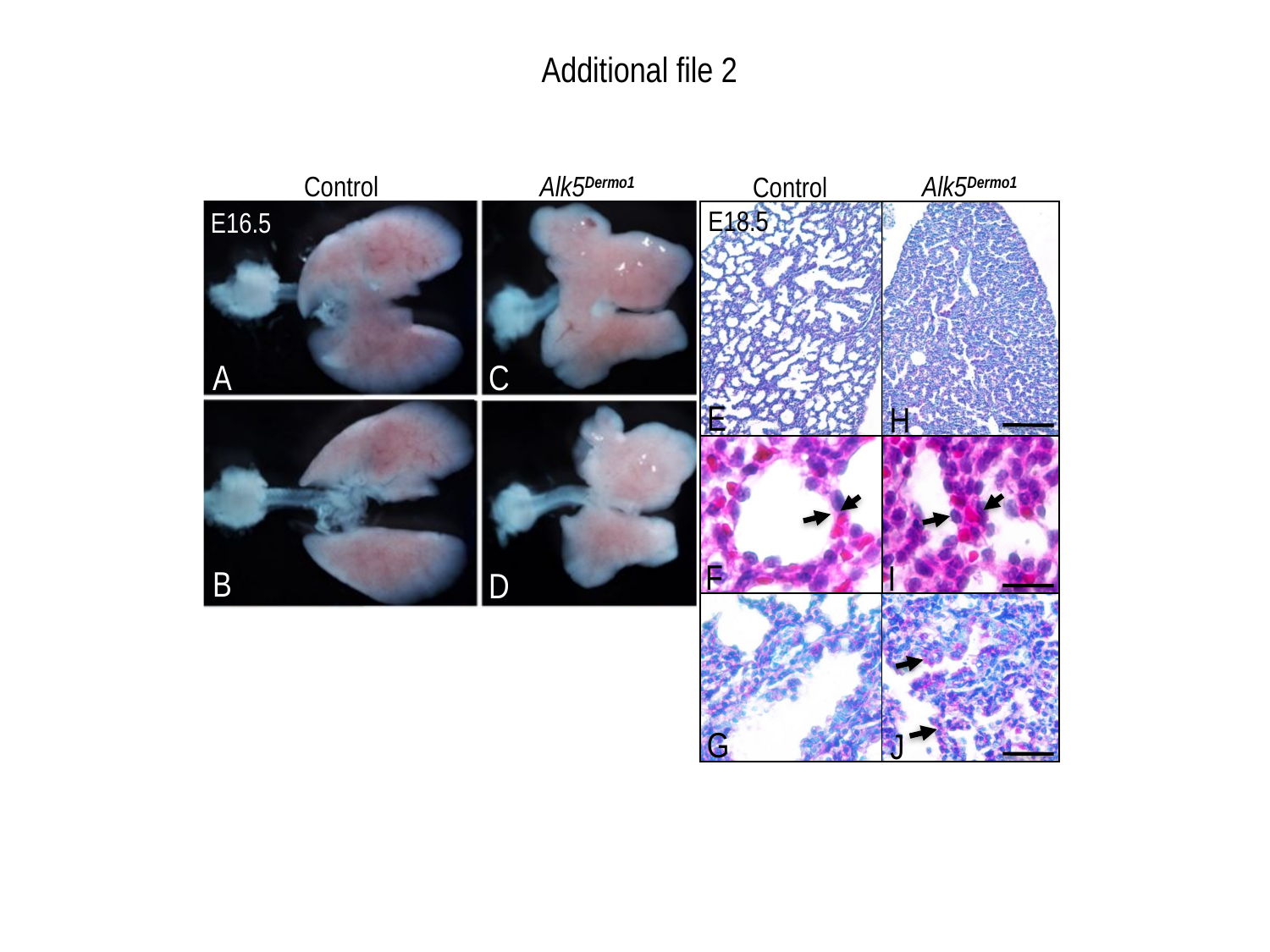

Additional file 2
Control
Alk5Dermo1
E16.5
A
C
B
D
E18.5
Control
Alk5Dermo1
E
H
F
I
G
J

Supplement: Additional file 2: — Mesodermal-specific Alk5 inactivation causes pulmonary hypoplasia. A–D. Gross morphology of E16.5 control (A and B) and Alk5 Dermo1 (C and D) lungs. E–J. PAS (E, H, G and J) and H&E (F and I) staining of E18.5 control (E–G) and Alk5 Dermo1 (H–J) lungs. Arrows in I indicate a thicker alveolar wall in Alk5 Dermo1 lungs; Arrows in J indicate robust PAS staining in Alk5 Dermo1 lungs. Scale bars: H = 100 μm; I = 10 μm, J = 20 μm. (PPTX 1736 kb) [file 12915_2016_242_MOESM2_ESM.pptx]

## Slide 1
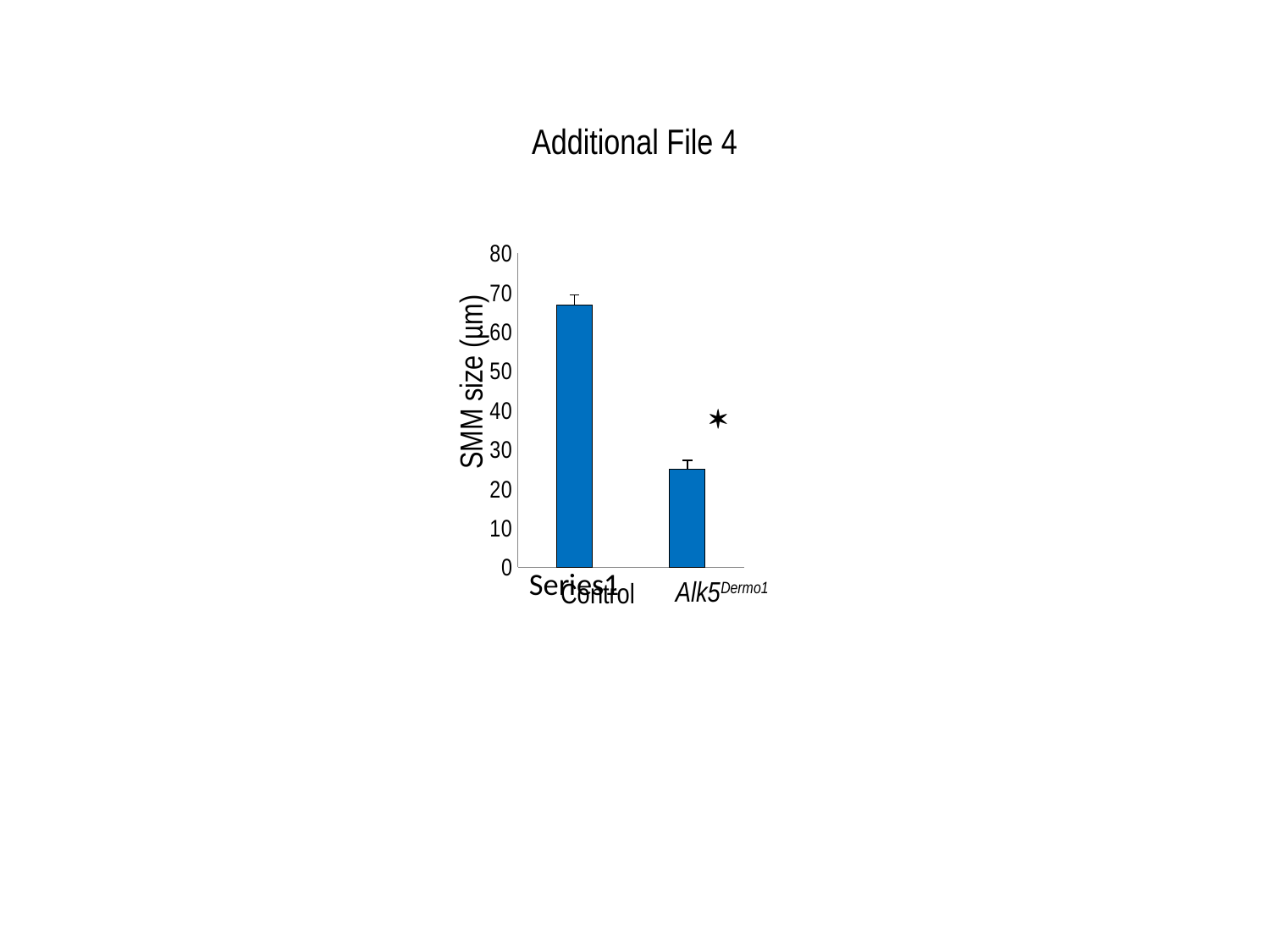

Additional File 4
### Chart
| Category | |
|---|---|
| | 66.8 |
| | 24.9 |SMM size (µm)
Alk5Dermo1
Control
*

Supplement: Additional file 4: — Quantification of sub-mesothelial mesenchyme (SMM) size. Hematoxylin and eosin-stained lung tissue sections were prepared and random images were collected under 40× objective from both control and Alk5 Dermo1 embryos at E12.5 and E13.5. The various dimensions that were measured included boundaries of the SMM compartments, as delineated on each photomicrograph, from mesothelium to the mesenchyme wrapped around the epithelium (SEM). The regions encompassing the SMM were measured in Photoshop by dividing each region at random of six points to manually calculate the length from mesothelium to SEM (μm). Sample size, n = 3 separate lung tissue sections. Error bars show standard deviation. *P <0.05. (PPTX 75 kb) [file 12915_2016_242_MOESM4_ESM.pptx]

## Slide 1
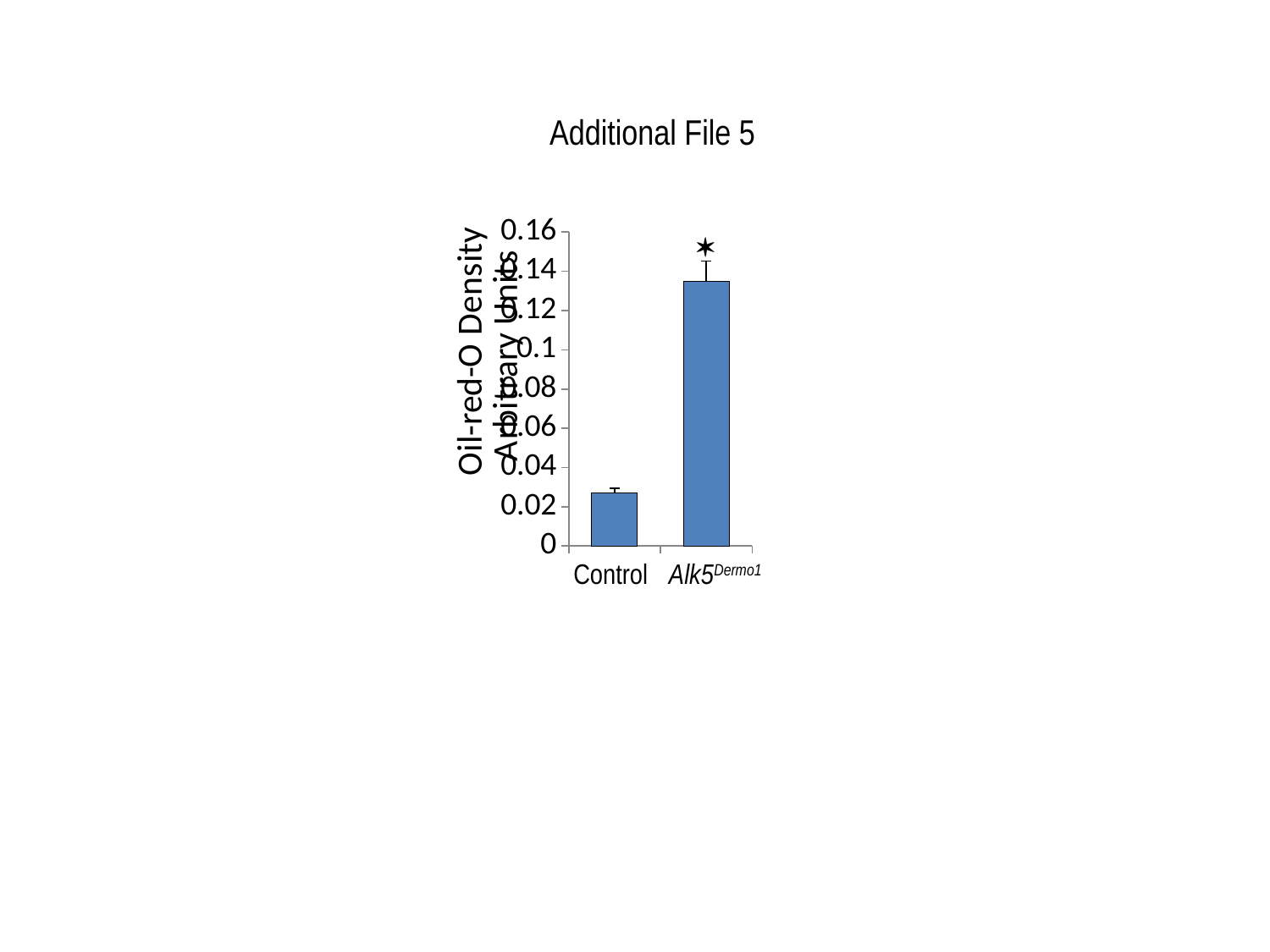

Additional File 5
*
### Chart
| Category | |
|---|---|
| Wild-type | 0.0269834137082147 |
| Alk5 KO | 0.134877146652741 |Control
Alk5Dermo1

Supplement: Additional file 5: — Quantification of Oil Red O storage. Lung tissue sections from E18.5 control and Alk5 Dermo1 lungs were analyzed by staining with Oil Red O for assessment of LIF differentiation. Oil Red O stained sections were viewed and random images were collected under 40× objective, and were analyzed by imaging using a previously described protocol [49], n = 3. The density of Oil Red O stained cells was then calculated and plotted as shown. Error bars show the standard deviation. *P <0.05 (PPTX 43 kb) [file 12915_2016_242_MOESM5_ESM.pptx]
