# Supplementary material for: Mesodermal ALK5 controls lung myofibroblast versus lipofibroblast cell fate
Source: BMC Biol. 2016 Mar 16;14:19. doi: 10.1186/s12915-016-0242-9 (PMC4793501; doi:10.1186/s12915-016-0242-9)
Supplement: Additional file 3: — Mesodermal Alk5 deficiency inhibits epithelial cell differentiation and disrupts vasculogenesis. A–H. Immunohistochemistry (IHC) for Acetylated-tubulin (A and E), CC10 (B and F), pro-SPC (C and G) and T1a (D and H) in E18.5 control and Alk5 Dermo1 lungs showing decreased expression of the latter genes in Alk5 Dermo1 lungs. I. Q-PCR confirmed inhibition of epithelial cell markers in Alk5 Dermo1 lungs, n = 2–9 pairs of separate lungs. J and L: IHC for GFP and FLK1 showing decreased GFP+/FLK1+ cells in E14.5 Alk5 Dermo1 lungs. K and M: IHC showed decreased CD34+ cells in E14.5 Alk5 Dermo1 lungs. N–Q. IHC showing decreased of PECAM1+ cells in E18.5 Alk5 Dermo1 lungs. Arrows indicate large blood vessels appearing intact. R. Quantitative PCR confirmed decreased Pecam1, Flk1, and Flt1 mRNAs in E13.5 mutant lungs. n = 3 pairs of separate lungs. Error bars show standard deviation. *P <0.05. Scale bars: H = 30 μm; M and Q = 20 μm. (PPTX 1560 kb) [file 12915_2016_242_MOESM3_ESM.pptx]

## Slide 1
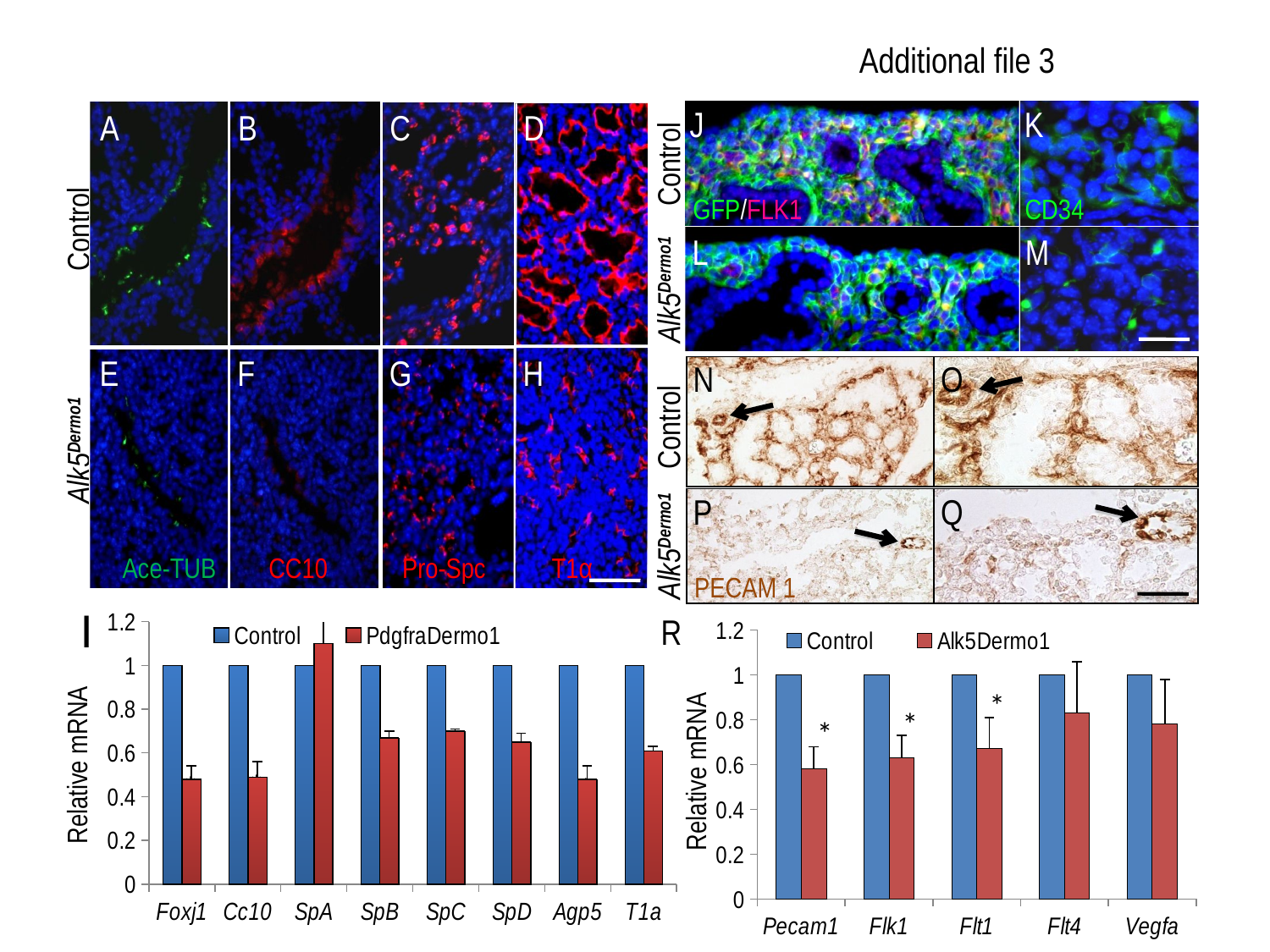

Additional file 3
J
K
A
B
C
D
Control
GFP/FLK1
CD34
Control
L
M
Alk5Dermo1
E
F
G
H
N
O
Control
Alk5Dermo1
P
Q
Alk5Dermo1
Ace-TUB
CC10
Pro-Spc
T1α
PECAM 1
I
### Chart
| Category | Control | PdgfraDermo1 |
|---|---|---|
| Foxj1 | 1.0 | 0.48 |
| Cc10 | 1.0 | 0.49 |
| SpA | 1.0 | 1.1 |
| SpB | 1.0 | 0.67 |
| SpC | 1.0 | 0.7 |
| SpD | 1.0 | 0.65 |
| Agp5 | 1.0 | 0.48 |
| T1a | 1.0 | 0.61 |R
### Chart
| Category | Control | Alk5Dermo1 |
|---|---|---|
| Pecam1 | 1.0 | 0.580000000000001 |
| Flk1 | 1.0 | 0.630000000000005 |
| Flt1 | 1.0 | 0.670000000000005 |
| Flt4 | 1.0 | 0.830000000000001 |
| Vegfa | 1.0 | 0.78 |*
*
*
Relative mRNA
*
Relative mRNA
*
*
*
*
*
