# Supplementary material for: Mesodermal ALK5 controls lung myofibroblast versus lipofibroblast cell fate
Source: BMC Biol. 2016 Mar 16;14:19. doi: 10.1186/s12915-016-0242-9 (PMC4793501; doi:10.1186/s12915-016-0242-9)
Supplement: Additional file 6: — Quantification of smooth muscle (SM) thickness and areas covered by smooth muscle in control and Pdgfrα Dermo1 lungs at E18.5. αSMA immunohistochemical stained lung sections were collected by random sampling using the 40× objective from three pairs of control and Pdgfrα Dermo1 mutant lungs at E18.5. The thickness and coverage of SM were quantified. A. Quantification of SM thickness. The arithmetic mean thickness of the SM cell layer was determined by volume of αSMApos compartment, measured by counting all points intercepting the airway epithelium and αSMApos compartment. n = 3. B. Quantification of SM coverage. The percentage of SM airway coverage was determined by assessing the percentage of the airway epithelium circumference in contact with number of αSMApos cells. n = 3. Error bars show the standard deviation. *P <0.05 (PPTX 54 kb) [file 12915_2016_242_MOESM6_ESM.pptx]

## Slide 1
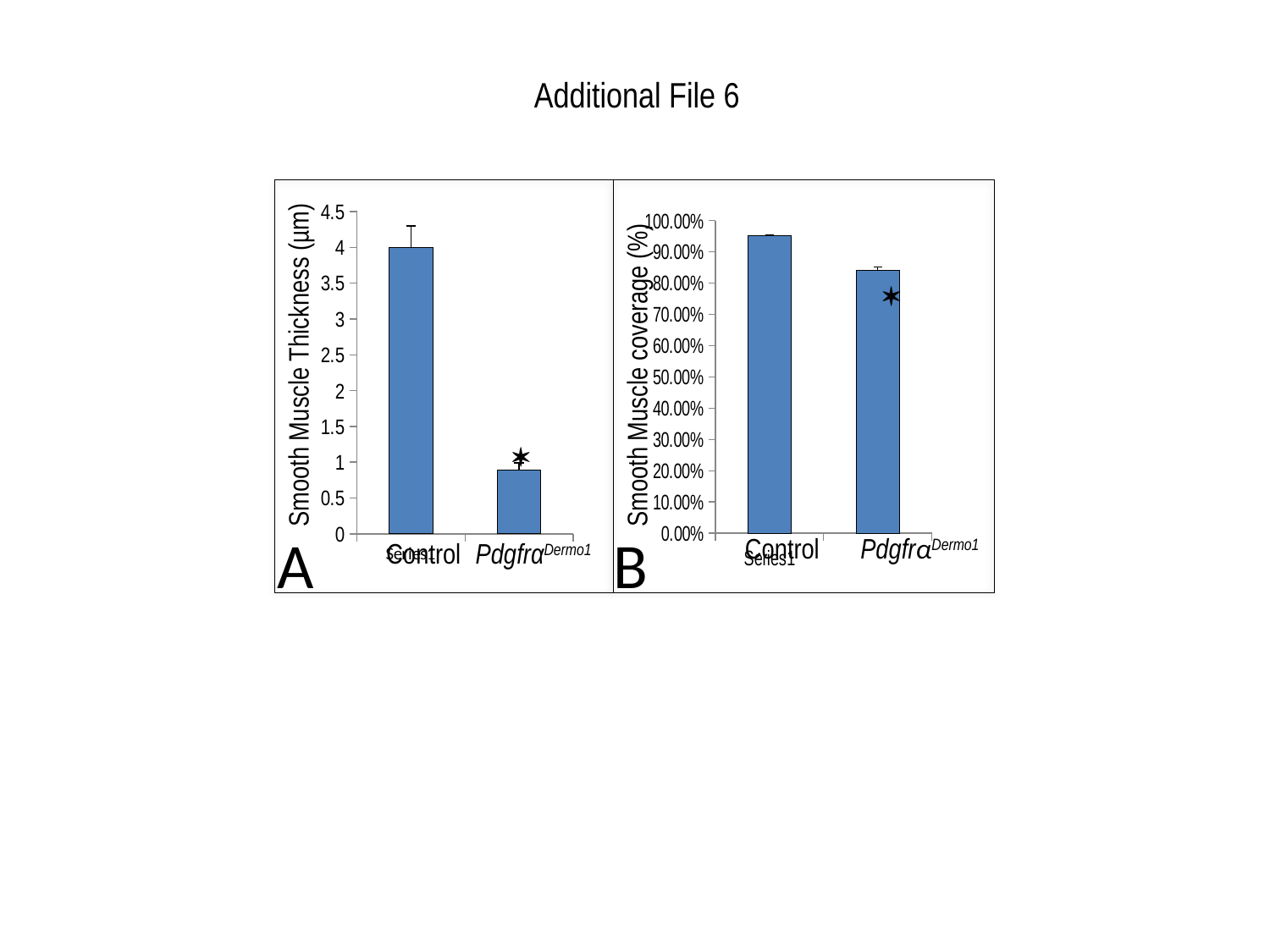

Additional File 6
### Chart
| Category | |
|---|---|
| | 4.0 |
| | 0.89 |Smooth Muscle Thickness (µm)
*
A
PdgfrαDermo1
Control
### Chart
| Category | |
|---|---|
| | 0.951 |
| | 0.8413 |*
Smooth Muscle coverage (%)
PdgfrαDermo1
Control
B
