# Supplementary material for: Mesodermal ALK5 controls lung myofibroblast versus lipofibroblast cell fate
Source: BMC Biol. 2016 Mar 16;14:19. doi: 10.1186/s12915-016-0242-9 (PMC4793501; doi:10.1186/s12915-016-0242-9)
Supplement: Additional file 7: — Mesodermal progenitor-specific deletion of Pdgfrα. A. Quantitative PCR (Q-PCR) showed PDGF signaling target genes are decreased, indicating an overall functional repression of PDGFA signaling pathway in E14.5 Pdgfra Dermo1 lungs. n = 2 pairs of separate lungs, repeated twice or thrice. B. Q-PCR showed repression of myofibroblast-related genes in Pdgfra Dermo1 lungs. n = 2 pairs of separate lungs, repeated thrice for each. Error bars show SEM. (PPTX 68 kb) [file 12915_2016_242_MOESM7_ESM.pptx]

## Slide 1
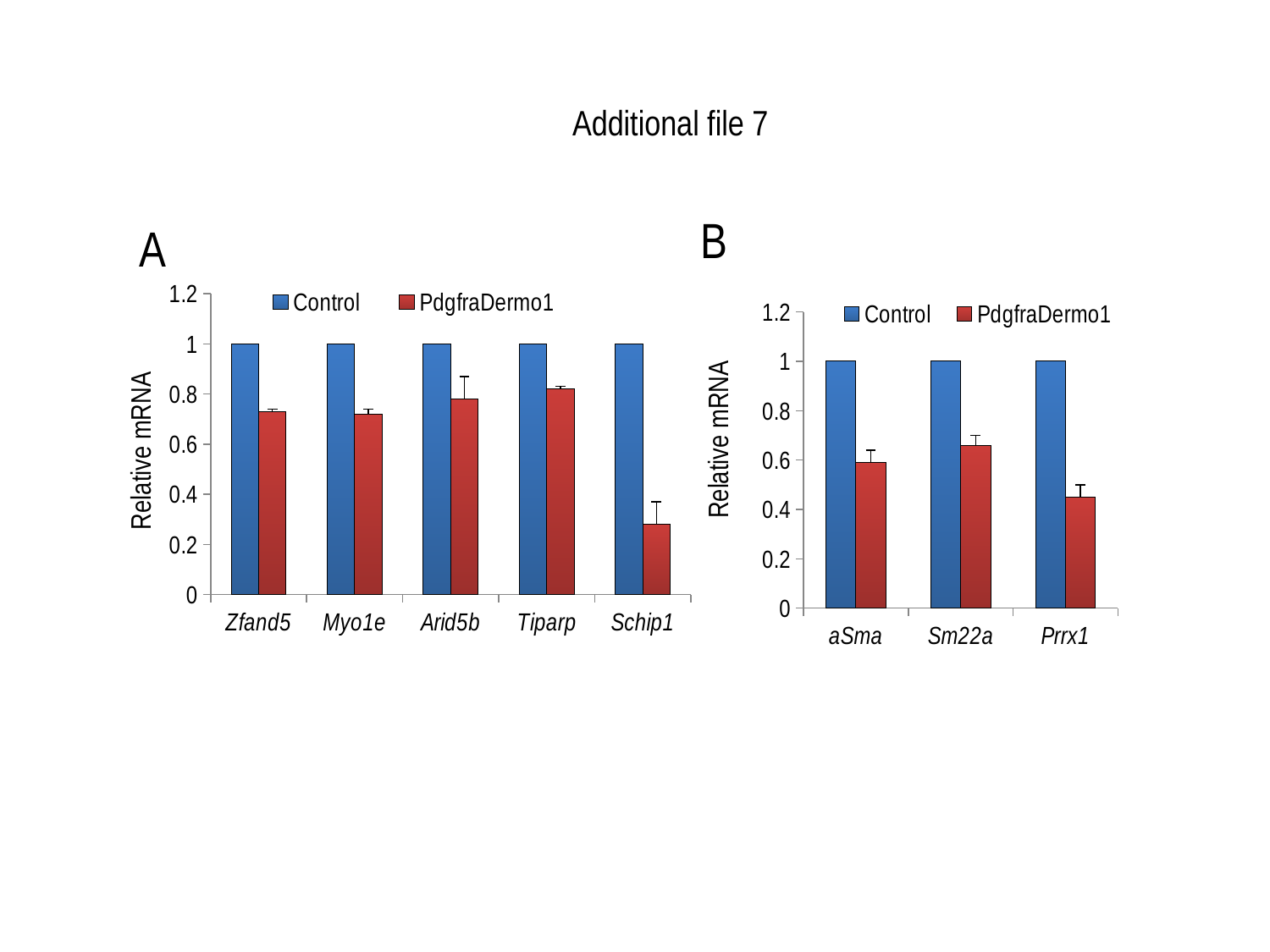

Additional file 7
B
A
### Chart
| Category | Control | PdgfraDermo1 |
|---|---|---|
| Zfand5 | 1.0 | 0.73 |
| Myo1e | 1.0 | 0.72 |
| Arid5b | 1.0 | 0.78 |
| Tiparp | 1.0 | 0.82 |
| Schip1 | 1.0 | 0.28 |
### Chart
| Category | Control | PdgfraDermo1 |
|---|---|---|
| aSma | 1.0 | 0.59 |
| Sm22a | 1.0 | 0.66 |
| Prrx1 | 1.0 | 0.45 |Relative mRNA
Relative mRNA
