# Supplementary material for: Mesodermal ALK5 controls lung myofibroblast versus lipofibroblast cell fate
Source: BMC Biol. 2016 Mar 16;14:19. doi: 10.1186/s12915-016-0242-9 (PMC4793501; doi:10.1186/s12915-016-0242-9)
Supplement: Additional file 8: — Mesodermal Alk5 deficiency increased cell apoptosis. The number of TUNELpos cells as determined by previously described methods [12]. A–D. TUNEL assay showed increased number of TUNELpos cells in the Alk5 Dermo1 lungs at E18.5. E. Quantification of the relative number of TUNELpos cells (green) per 4,000 total cells. Error bars show standard deviation. *P <0.05. (PPTX 794 kb) [file 12915_2016_242_MOESM8_ESM.pptx]

## Slide 1
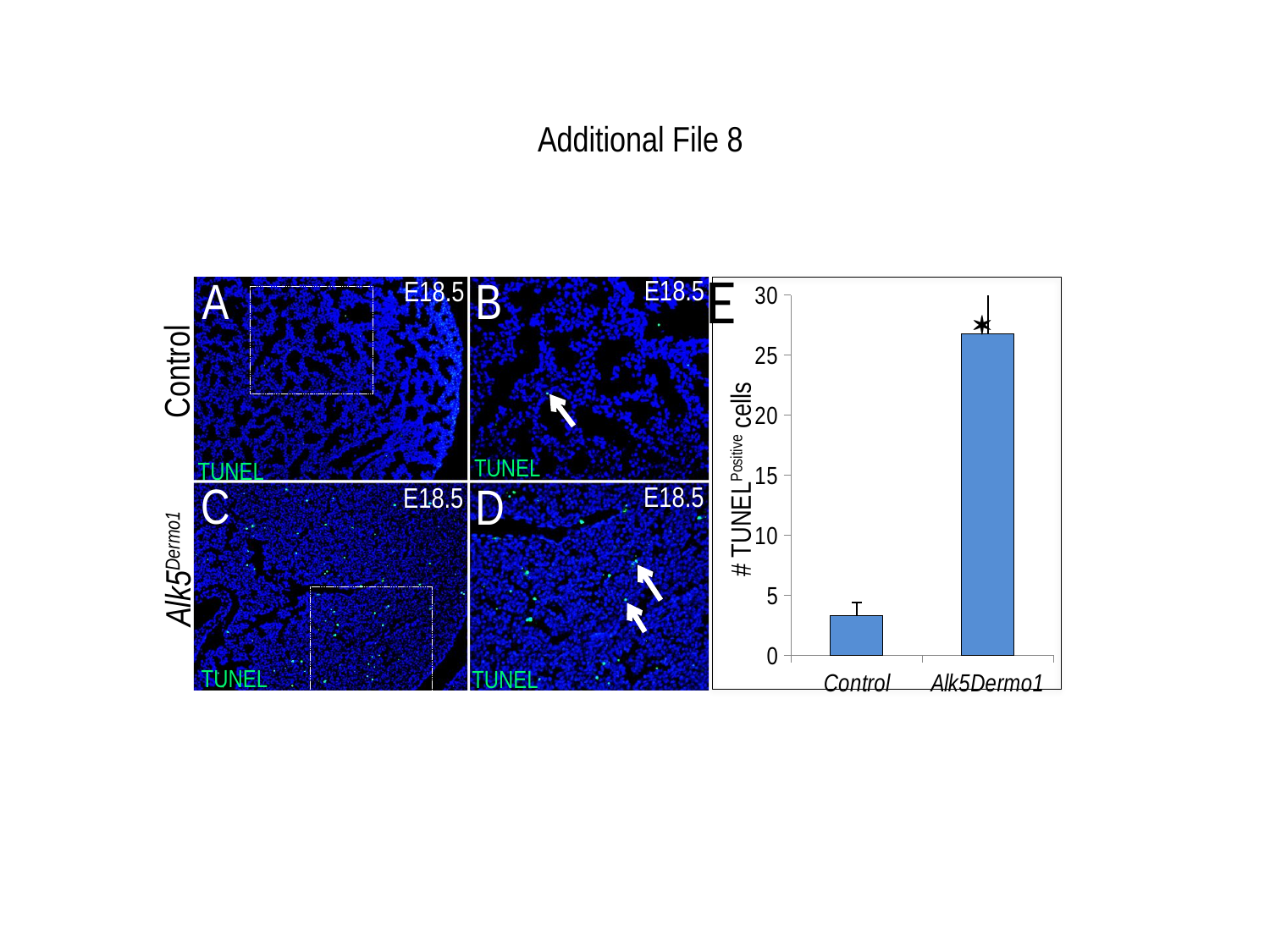

Additional File 8
E
### Chart
| Category | |
|---|---|
| Control | 3.29 |
| Alk5Dermo1 | 26.8 |# TUNELPositive cells
*
A
B
E18.5
E18.5
Control
TUNEL
TUNEL
C
D
E18.5
E18.5
Alk5Dermo1
TUNEL
TUNEL
