# Supplementary material for: Mesodermal ALK5 controls lung myofibroblast versus lipofibroblast cell fate
Source: BMC Biol. 2016 Mar 16;14:19. doi: 10.1186/s12915-016-0242-9 (PMC4793501; doi:10.1186/s12915-016-0242-9)
Supplement: Additional file 10: — Primary antibodies used in western blots and immunohistochemistry. (DOC 37 kb) [file 12915_2016_242_MOESM10_ESM.doc]

| **Additional file 10. Primary antibodies used in western blots and IHC** | | |
| --- | --- | --- |
| **Name** | **Type** | **Source** |
| ALK5 | Rabbit polyclonal | Abcam |
| PAI-1 | Rabbit polyclonal | Abcam |
| PPARγ | Mouse monoclonal | Sigma |
| ADRP | Mouse monoclonal | Abcam |
| PECAM1 | Mouse monoclonal | Sigma |
| Ace-tubulin | Mouse monoclonal | Biogenex |
| β-ACTIN | Rabbit polyclonal | Abcam |
| CC10 | Goat polyclonal | Santa Cruz |
| Pro-SPC | Mouse monoclonal | 7-Hills |
| T1a | Rabbit polyclonal | Abcam |
| p-SMAD2 | Mouse monoclonal | Cell Signaling Technology |
| p-ERK | Rabbit polyclonal | Cell Signaling Technology |
| PDGFRa | Rabbit polyclonal | Cell Signaling Technology |
| p-AKT | Rabbit polyclonal | Cell Signaling Technology |
| Phospho-Histone-H3 | Mouse monoclonal | Cell Signaling Technology |
| αSMA | Mouse monoclonal | Sigma |
| FABP4 | Mouse monoclonal | Santa Cruz |
| CEBPα | Rabbit polyclonal | Santa Cruz |
